# Boosting Drug Discovery: Expanding the Applicability of Fragment Dissolved Molecular Dynamics to Accelerate Binding Mode Elucidation

**DOI:** 10.1021/acs.jcim.5c02122

**Published:** 2025-11-24

**Authors:** Maria Nuria Peralta-Moreno, José M. Granadino-Roldán, Maria Santos Tomas, Jaime Rubio-Martinez

**Affiliations:** † Departament de Ciència dels Materials i Química Física, Universitat de Barcelona (UB) and the Institut de Química Teòrica i Computacional (IQTCUB), Martí i Franqués 1-11, 08028 Barcelona, Spain; ‡ Departamento de Química Física y Analítica, 16747 Universidad de Jaén, Campus “Las Lagunillas” s/n, 23071 Jaén, Spain; § Department of Architecture Technology, 16767 Universitat Politècnica de Catalunya, Av. Diagonal 649, 08028 Barcelona, Spain

## Abstract

The use of small organic molecules has become one of the most popular strategies in computer-aided drug design (CADD) to facilitate the identification of potential drug-like compounds in the early stages of drug development. In this scenario, novel computational approaches such as the use of the fragment dissolved molecular dynamics (fdMD) methodology emerged as a new framework for the modeling of ligand–receptor interactions. Consisting of molecular dynamics (MD) simulations of the target protein solvated with multiple copies of the same fragment, the original approach is able to identify the most favorable binding site for the system studied in a reasonable simulation time scale (0.2–1 μs). In the present work, we have introduced the use of Gaussian accelerated molecular dynamics (GaMD) to facilitate system exploration, accelerate binding site identification and additionally enhance binding mode elucidation. For this purpose, up to 12 different systems with crystallographic information available have been employed for validation.

## Graphical abstract

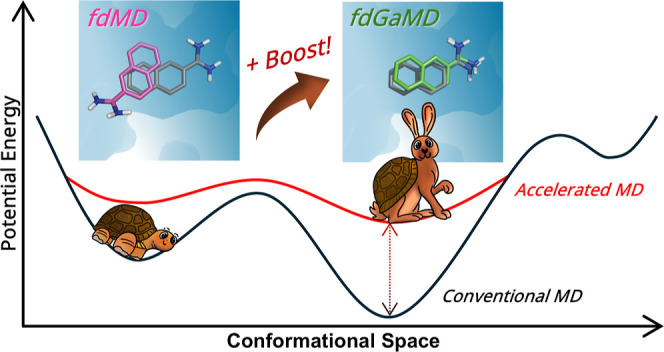

## Introduction

In drug discovery, automated testing of large compound libraries for high-throughput screening (HTS) is typically limited to the chemical space of drug-like molecules.^1,2^ As a means to efficiently explore ligand–receptor interactions, a different conception of drug design called fragment-based drug discovery (FBDD) emerged as one of the most promising strategies in the search for more specific therapies.^3–7^ In this approach, protein interactions are deeply explored using small organic molecules that will further serve as scaffolds for hit-to-lead optimization into more specific and active drug candidates.^4^

Common limitations, such as false negatives and positives arising from the inherent constraints of detection methods or the lack of detailed knowledge of the fragment–target interactions, often prevent the process from finishing successfully. Overcoming these challenges typically require an integrative strategy that combines multiple computational and experimental technologies.^8–10^ For example, combining molecular docking or molecular dynamics (MD) techniques with experimental assays, particularly in the early stages of the drug discovery process, has significantly enhanced the ability to predict protein–ligand interactions in the search of new drug candidate molecules.^11–15^

Alternatively, approaches based on multiple solvent MD simulations emerged as new strategies to unravel potential druggable hotspots and allosteric sites of the targeted protein,^16–23^ as done before in crystal structures.^24^ Similarly, instead of solvents, different applications started to involve a high concentration of molecular probes or small ligands to increase system exploration with more specific interactions.^14,25–30^ This type of MD simulations was initially limited to up to a reduced number of fragment copies due to ligand aggregation. In this scenario, the fragment-dissolved molecular dynamics (fdMD) approach emerged as a promising solution to enhance sampling efficiency by introducing a Lennard-Jones repulsive term into the ligand parameters.^31^ This systematic and semiautomatic strategy significantly reduced the computational costs of producing multiple simulations and overcame the typical limitations of multiple copies studies.

Compared to other methodologies,^32^ a single global fdMD simulation can generate as many individual target–ligand trajectories as the number of fragment copies in the simulation box. As a result, protein exploration significantly enhances as the probability of finding druggable spots is increased by the use of multiple ligands. Then, through the fdMD analysis protocol, it is possible to identify the binding site with the most interesting and favorable interactions. However, in some cases, spurious results may hinder the selection process. Additionally, conformational changes may not occur in a computationally reasonable time with conventional MD (cMD), impeding the fragment from adopting the most favorable binding mode.

So far, many efforts have been undertaken to address the common challenges associated with conventional single protein–ligand molecular dynamics simulations. For example, the use of Gaussian accelerated molecular dynamics (GaMD),^33,34^ ligand-oriented MD simulations,^35–37^ or steered MD-based approaches (SMD),^38–40^ among others, have provided alternative solutions to increase the conformational sampling in complex systems where long cMD are necessary to observe the process under study.

With the ultimate goal of not only predicting binding sites in the absence of experimental data but also elucidating the most favorable binding mode for any given system, this work introduces the integration of Gaussian accelerated molecular dynamics (GaMD) into the original fragment-dissolved molecular dynamics (fdMD) framework. By doing so, we aim to combine the robust sampling of employing MD simulations with multiple copies of the same ligand and the potential of GaMD to enhance conformational exploration and, moreover, avoid the detection of low-affinity spurious sites to facilitate binding mode elucidation. For this purpose, diverse protein–fragment systems with available crystallographic information have been employed for validation. As a result, we propose the fragment dissolved Gaussian accelerated molecular dynamics (fdGaMD) approach as an interesting and promising alternative strategy in drug design for binding mode elucidation.

## Computational Methods

### Fragment Dissolved Molecular Dynamics (fdMD)

Devoted to binding site identification, fdMD is a computational method originally designed to facilitate system exploration using conventional molecular dynamics simulations of the target protein solvated with multiple copies of the same fragment in a TIP3P water medium.^31,41^ Aimed to identify and rank the most favorable binding sites of the studied fragment toward a certain protein, the method consists in a systematic and semiautomatic workflow in which systems are prepared,^42,43^ optimized,^44^ parametrized,^45,46^ built and minimized,^47^ to perform several independent fdMD simulations at 300 K in the *NVT* ensemble under PBC conditions.^48^ Then, for each of the replicates, an individual trajectory analysis is performed in which a set of descriptors are computed and evaluated to identify the binding site exhibiting the best ligand–receptor interactions for the studied system.^31^

Moreover, fdMD originally proposed the introduction of a repulsive term in the central atom of the fragment to address one of the most common limitations of FBDD in computational studies involving the use of multiple copies.^14,26,30,32^ Consequently, ligand aggregation during the simulation is avoided thanks to the addition of a Lennard-Jones repulsive term (with the attractive part set to zero) between the central heteroatom of the ligands, parametrized as **C**99/**N**99/**O**99/**S**99 in the topology, accordingly.

The approach has demonstrated to be able to identify the most favorable binding site without prior crystallographic knowledge.^31^ Still, different scenarios such as the presence of false positives or fragments exhibiting low affinities can result in spurious results that can hinder the selection process for more complex systems. Given the advantages of the proposed methodology and the challenges it addresses, this work introduces an alternative approach to the original method through the incorporation of Gaussian accelerated molecular dynamics (GaMD).^34^

### Gaussian Accelerated Molecular Dynamics (GaMD)

Conventional molecular dynamics (cMD) simulations are extensively used to describe the behavior of a molecular system and predict its evolution over time, providing a dynamic model of a molecular system.^49^ By the integration of Newton’s equations of motion, the conformational space can be explored and studied within a typical simulation time scale framework. In some cases, though, it is difficult to capture information due to the complexity of the system itself. Alternatively, thanks to the adaptative potential boost introduced with Gaussian accelerated molecular dynamics (GaMD) simulations, it can be possible to overcome some of these limitations habitually associated with the presence of high energetic barriers.^34^

The potential applied (Δ*U*), either to adaptatively boost both the system’s potential and/or the dihedral energies (*U*), is based and obtained through harmonic functions and Gaussian distribution principles (eq 1). The harmonic force constant (*k*), defined in eq 2, plays a crucial role in the magnitude of the potential boost applied. With *U*_max_ and *U*_min_ being the maximum and minimum potential energy sampled, the scaling factor *k*_0_ corresponds to an optimizable value in the [0, 1] range. Knowing that *k*_0_ = 1 corresponds to the maximum potential to be applied, the optimization of the scaling factor and related parameters is essential prior to any simulation. Parameters should permit an enhanced unconstrained conformational exploration of the system, while preventing the system from destabilizing. The boost then will only be applied when the potential energy of the system is lower than a pre-established threshold (*E*), focused on favoring transitions between different energetic metastable states that otherwise will be difficult to access (eq 3).^33^ Accordingly, the potential energy surface is smoothed (*U*′), enabling a more efficient system exploration and favoring conformational changes to occur (Figure 1). However, because of this potential energy surface modification, a reweighting process must be done to recover the unbiased probabilities needed to calculate some properties. Furthermore, although the method itself controls the amount of boosting that is introduced, it is important to check that it does not introduce undesirable structural changes into the system.

1 $$\Delta U\left(r\right)=\frac{1}{2}k{\left(E-U\left(r\right)\right)}^{2}$$

2 $$k\equiv k_{0}\left(\frac{1}{U_{\max}-U_{\min}}\right)$$

3 $$U^{\prime }\left(r\right)=\{\begin{array}{ll} U\left(r\right) & U\left(r\right)\geq E\\ U\left(r\right)+\Delta U\left(r\right) & U\left(r\right)<E \end{array}$$

**Figure 1:**
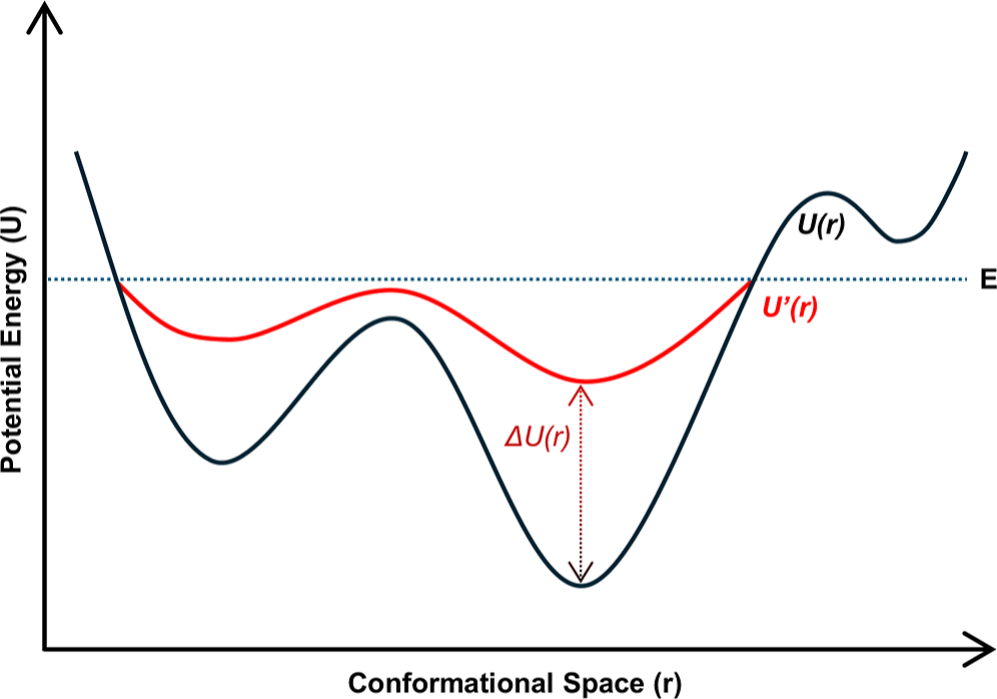
Illustrative 2D representation of a potential energy surface (*U*, PES) smoothed (*U*′) by the application of a Gaussian accelerated potential energy boost (Δ*U*), as implemented in GaMD simulations by Miao et al.^33^ The enhanced sampling approach thus permits a faster and efficient exploration due to the reduction of the energetic barriers between metastable states.

### Fragment Dissolved Gaussian Accelerated Molecular Dynamics (fdGaMD)

As described above, the fragment dissolved Gaussian accelerated molecular dynamics (fdGaMD) approach aims to improve conformational sampling and binding site exploration, thereby enabling the identification of optimal interactions. Given the importance of binding mode elucidation in future optimizations of the fragment into a larger drug-like compound (hit-to-lead process), it is essential to explore the system in the search of potential druggable sites and be able to select those exhibiting the most promising results. By the introduction of GaMD, the potential energy surface of the system is smoothened, lowering energetic barriers associated with conformational changes to escape local minima, as well as dismissing energetically less favorable interactions that potentially could result in spurious results. Accordingly, fdGaMD integrates GaMD-based enhanced sampling with multicopy fragment MD simulations in a systematic, semiautomated protocol designed to accurately determine the most favorable binding mode.

During the first step, receptor and fragment structures are prepared and minimized. Next, the target protein is solvated by the addition of multiple copies of the ligand box (Figure 2). As a result, the final simulation box will contain the target protein and the fragments, all in a TIP3P water molecules, with the necessary counterions to neutralize the system, if required. Subsequently, the system undergoes minimization, heating, and equilibration before performing a minimum of four 400 ns fdGaMD replicates to enhance conformational sampling and protein exploration, implemented in the AMBER molecular dynamics package.^47,50,51^ More detailed information is included in “System Preparation & GaMD Optimization for fdGaMD Simulations” subsection.

**Figure 2:**
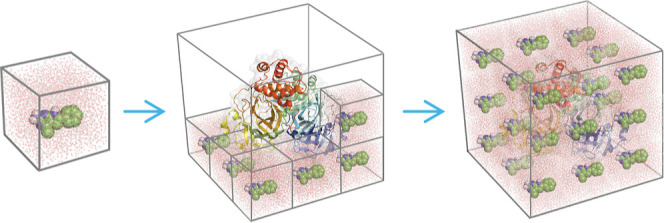
Illustrative 3D representation of a fdMD system simulation box construction process using the LEaP module of Amber22 software.^47^ Depicted from left to right, a pre-equilibrated ligand box of the fragment in TIP3P water molecules is first prepared and then used to solvate the target protein by adding multiple copies of this box, yielding the complete system for subsequent fdMD/fdGaMD simulations.

For the analysis, each independent fdMD simulation with multiple copies of the fragment is striped into individual ligand–receptor trajectories, where water molecules and counterions are removed to focus on evaluating ligand–receptor interactions and reduce the associated computational cost. For this purpose, a set of descriptors is computed and evaluated for those individual ligand trajectories evaluating interactions toward the target protein. Then, for each system, a classification per binding site identified is obtained for final evaluation and subsequent selection of the most favorable site and binding mode, as described thoroughly in the Computational Methods subsection “Trajectory & Binding Analysis”. Alternatively, diverse and varied analysis can be performed like the MDmix method.^22^ Unfortunately, these approaches are based on density population analyses, which leads to the loss of dynamic binding information. Consequently, within the fdGaMD protocol presented (Figure 3), we adopted an analysis focused on individual ligand–receptor trajectories, which we consider to be key for understanding and accurately capturing dynamic binding events.

**Figure 3:**
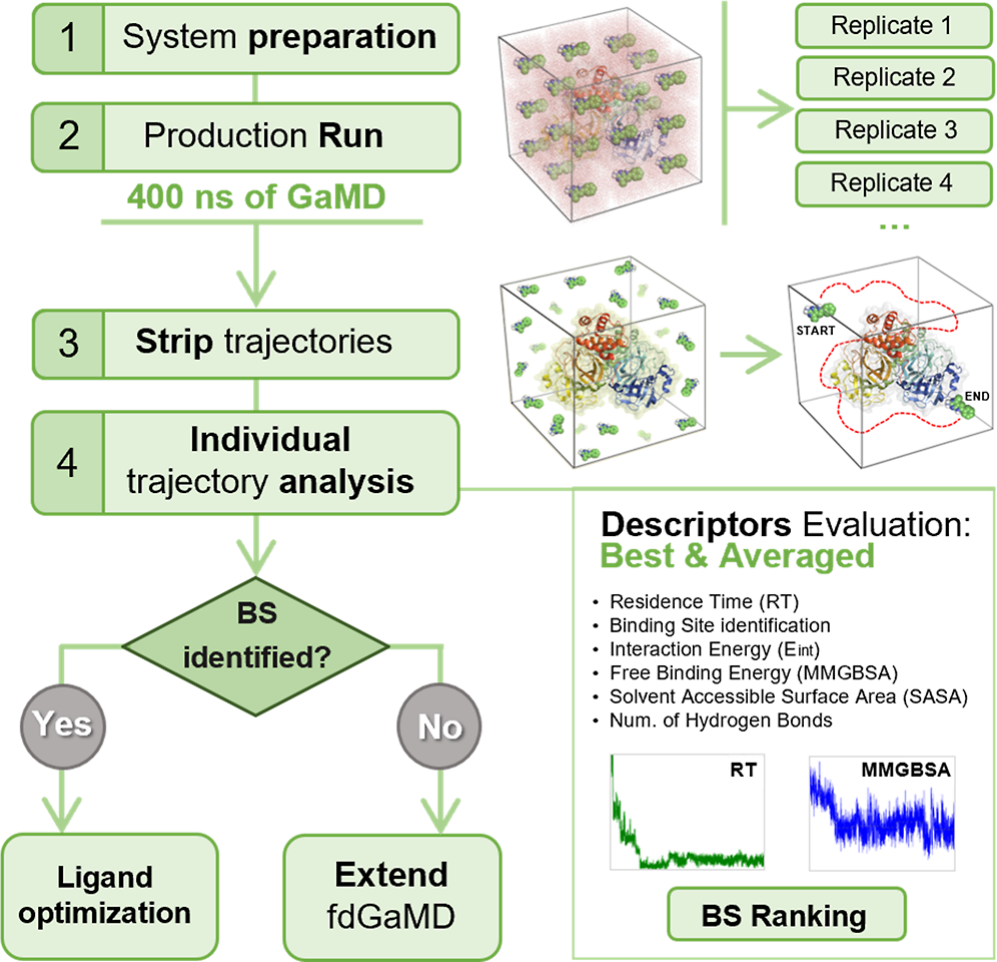
Schematic representation of the complete semiautomatic fdGaMD workflow, highlighting the main steps of the approach consisting of: (1) preparation of the fdGaMD solvated simulation system box, and subsequent minimization, heating and equilibration; (2) production run of multiple independent fdGaMD replicates; (3) separation of the individual trajectories from the global fdGaMD simulation of multiple ligand copies; and (4) descriptor-based final analysis to assess binding affinities and select the most favorable site of interest.

### Validation: Selection of the Studied Systems

Originally, the fdMD approach was validated using a diverse selection of protein–ligand complexes to demonstrate its applicability to systems without prior knowledge of the experimental binding site or affinity.^31^ Similarly, since the present work aims to propose the introduction of GaMD simulations to the main methodology, the approach has been subjected to a validation process. For this purpose, 12 protein–fragment complexes with available crystallographic information, whose fragment molecular weight did not exceed 300 g/mol, were selected and classified into 3 different sets to evaluate the robustness of the methodology, focusing not only on binding site identification but also on binding mode elucidation (Table S1).

To establish a comparable baseline to the use of cMD in fdMD simulations, the myeloid cell leukemia 1 (MCL-1) protein and two studied fragments (class I & class II), were selected from previous fdMD validation as the initial data set (set-I) to be evaluated.^31^ In this case, only the X-ray structure of the protein (PDB ID: 4HW3), complexed with a ligand derived from the linking of class I (C1G) and class II (19G) fragments, was available, as shown in Figure S1.^52^ Despite not being determined crystallographically, both fragments are known to independently inhibit MCL-1 protein, with inhibition constant *K*_i_ values of 131 mM and 60 μM, respectively.^52^ Accordingly, following the procedure used to validate fdMD, class I and class II fragments were independently examined to evaluate fdGaMD performance. For both systems, the apo form of the MCL-1 target protein (with PDB ID 4OQ5) was employed.^53^

To evaluate the effect of the acceleration introduced on binding site identification, the same urokinase target protein—known to form complexes with three different fragments of increasing experimental binding affinities—was selected to constitute the second evaluation set (set-II). All three small fragments (BEN, 2UP, and 6UP) bind to the orthosteric binding site of the urokinase-type plasminogen activator (uPA) target protein, as determined by their crystal structures with PDB IDs 1F5K, 4FU8, and 4FUD, respectively.^54^ Table S1 illustrates that a fragment’s binding affinity and ability to form interactions influence complex stability, making weak fragments more likely to dissociate than optimized, larger ligands.

For the last set of systems to evaluate (set-III), we selected a variety of protein–ligand complexes with crystallographic information available in a wide range of experimental binding affinities between 10^3^ and 10^0^ μM, with diverse targets such as platelet-activating factor acetylhydrolase (Lp-PLA2), mitogen-activated protein kinase 14 (MAPK14) or activated coagulation factor (FXIa).^55–58^ Moreover, we wanted to evaluate the accuracy of the fdGaMD approach in binding site prediction and binding mode elucidation of larger and more affine fragments. For this purpose, a tyrosine-protein Janus kinase (JAK-2; with PDB ID 3E63) and an α-phosphoinositide dependent kinase 1 (PDK-1) system (with PDB structure 3NUN) were selected and included in the last validation set.^59,60^ Besides, a small organic molecule known to bound to the major urinary protein I (MUP-I) with no reported experimental binding assays (PDB ID 1I06),^61^ was also included to test the robustness of the approach in reproducing its binding mode without any prior knowledge about its affinity.

### System Preparation & GaMD Optimization for fdGaMD Simulations

As described in the fdMD workflow by Privat et al.,^31^ systems were prepared and optimized from the crystallographic structures available at the protein data bank (PDB).^42,62^ Ligand charges were computed with the restrained electrostatic potential RESP model at the HF/6-31G­(d,p) level and the repulsive Lennard-Jones potential term was introduced, by means of the Antechamber and ParmEd AMBER modules, respectively.^44,47^ Ligand and protein parameters were respectively obtained with the generalized AMBER force field (GAFF2) and the ff14SB AMBER force field.^45,46^

Following the main protocol, the studied fragments were solvated with TIP3P water molecules using the LEaP module of Amber22 (Figure 2).^47^ The size of the ligand box is determined by the minimum distance from the edge of the simulation box to the ligand (*d*_min_), which by default is set to 15 Å. Then, minimized solvated ligand boxes were subsequently added within 20 Å of the receptor. By doing so, during the preparation of either the ligand or the protein simulation boxes, molecules with any atom closer to 1 Å of the ligand or protein atoms, respectively, were removed to avoid steric clashes and submitted to minimization, heating and equilibration protocols as previously described. Dimensions of the simulation box varied depending on the ligand and protein sizes, as well as the number of ligand copies for each of the studied systems. This information is available in Table S1.

As an improvement to the fdMD protocol for optimal ligand distribution during simulation box preparation, both the protein and ligand were aligned along their respective principal axes to minimize rotational bias and enable efficient solvation.^63^ This has meant a significant reduction in the number of water molecules used to solvate the system and, thanks to this new efficient arrangement, a slight reduction in the computational cost associated with the simulations. Thus, as a first step in the preparation of the simulation boxes prior to solvation, fragments were oriented in their principal axes using CppTraj module of AmberTools (Figure S9).^47^ Then, the same strategy was used for the target protein prior solvation.

After preparing the system simulation box, prior to the production run, the system must be subjected to a minimization and equilibration run to adapt the system to dynamic conditions. According to an interesting discussion about GaMD,^64^ the system must be properly equilibrated before running any GaMD simulation.^34^ It was suggested by Wang to consider the system size dependence in the equilibration for the potential statistics in GaMD simulations, with special focus on ensuring that the total and dihedral harmonic force weight parameters *k*_0_ reach the unity (see Gaussian Accelerated Molecular Dynamics (GaMD) section for clarity). Subsequently, according to his suggestion for AMBER GaMD simulations, the *ntave* variable was set as four times the number of total atoms in the system, and variables *ntcmdprep*, *ntebprep* and *ntcmd* as following in eqs 4–7, to ensure parameter optimization for the application of the maximum boost potential to every system.

4 $$ntave=4N_{atoms}$$

5 ntcmdprep=2ntave

6 ntebprep=2ntave

7 ntcm=5ntave

where *ntave*, *ntcmdprep*, *ntebprep* and *ntcmd* variables correspond to the frequency in which the running average of potential energy is updated, the number of steps needed to estimate the initial potential values to determine the boost potential, the number of steps in which the GaMD boost is introduced and the total number or production steps, respectively.

### Descriptors

Descriptors play a crucial role quantitatively comparing and evaluating similarities, specific properties or the behavior of any molecular system.^65^ In our case, since the fdGaMD methodology is aimed at identifying fragments exhibiting the best binding toward a target protein, we mainly focused on interaction descriptors for the subsequent trajectory and binding analysis. The calculation of residence time (RT), intermolecular free binding energy contributions (*E*_inter_), solvent accessible surface area (SASA) of the exposed ligand, molecular mechanics generalized-Born surface area (MMGBSA) free binding energy, and number of hydrogen bonds, is performed for each analyzed ligand trajectory. To support the interpretation of the results, this section aims to provide further insight into the descriptors selected for the trajectory analysis.

It is important to clarify that RT is defined as the time during which the ligand remains stably bound throughout the entire MD simulation, rather than the time required for ligand unbinding, which is the conventional definition. Therefore, RT is not related to the dissociation constant (*K*_d_) nor does it possess direct physical significance, serving instead as a descriptor metric that adds an additional criterion for identifying the most frequently predicted binding.

Calculation of free binding energies (Δ*G*_bind_) is an essential step for stability evaluation of ligand–receptor interactions and system behavior (eq 8). By assessing the main energetic contributing terms, such as the interaction energy (Δ*E*_MM_) or the solvation effect (Δ*G*_solv_) (eq 9), we can obtain more insight into the nature of the complex formation. As part of the interaction energy, van der Waals (*E*_vdW_) and electrostatic interactions (*E*_elec_) are computed for the descriptor analysis with the Amber22 CppTraj module,^47^ to account for intermolecular nonbonded (*E*_inter_) contributions in ligand–receptor complex formation (eqs 10–12).

8 $${\Delta G}_{bind}=G_{complex}-\left(G_{receptor}+G_{ligand}\right)$$

9 $${\Delta G}_{bind}=\Delta H-T\cdot \Delta S={\Delta E}_{MM}+{\Delta G}_{solv}-T\cdot \Delta S$$

10 $${\Delta E}_{MM}={\Delta E}_{intra}+{\Delta E}_{inter}$$

11 $$E_{intra}=E_{bonds}+E_{angles}+E_{torsions}+E_{improper}$$

12 $$E_{inter}=E_{vdW}+E_{elec}$$

To ensure robustness and cross-validation of binding estimations, the MMGBSA end-point approach was also employed for the calculation of the free binding energy (Δ*G*_bind_) of the complex.^66^ While the calculation of the nonbonded interaction energy contribution (Δ*E*_inter_) provided an estimation of van der Waals (vdW; *E*_vdW_) and electrostatic (*E*_elec_) interactions, MMGBSA can provide a better description of binding free energies through the introduction of solvation effects (Δ*G*_solv_) containing the polar contribution of the generalized Born (GB; Δ*G*_GB_) implicit solvent model and the surface area (SA; Δ*G*_SA_) nonpolar contribution (eqs 13–15). Using both approaches allows for comparison between a direct energy decomposition based on nonbonded intermolecular interactions of the complex in the vacuum to account for vdW and electrostatic ligand–receptor contributions, and a more detailed and comprehensive semiphysical approach to incorporate solvation effect through the GB model and enable a more comprehensive understanding of ligand binding, especially for systems involving charges.^67^ Hence, as a measure of validation of the obtained results, agreement or deviation between both approaches can highlight characteristics (such as ligand charge) or help interpret stability and solvation effects. For the purpose, the MMGBSA.py python program was employed.^68^

13 $${\Delta G}_{solv}={\Delta G}_{polar}+{\Delta G}_{non‐polar}$$

14 $${\Delta G}_{bind}={\Delta E}_{MM}+{\Delta G}_{solv}^{polar}+{\Delta G}_{solv}^{non‐polar}-T\cdot \Delta S$$

15 $${\Delta G}_{bind}^{MMGBSA}={\Delta E}_{MM}+{\Delta G}_{GB}+{\Delta G}_{SA}-T\cdot \Delta S$$

We used the one-trajectory approximation, where Δ*E*_intra_ = 0, assuming the entropic contribution to be constant. Since we are working with fragments, which typically present few degrees of freedom, we can expect the entropic contribution to be quite similar within the same fragment. On the other hand, it is well-known that MMGBSA cannot reproduce the absolute binding free energy, although the relative values are very reasonable. In our context, we use this value as another descriptor contributing to the selection of the best binding site/mode, but it is not definitive on its own. Instead, we use all descriptors together, in a consensus approach; that is, the most voted binding pose is selected as the most probable/favorable. This strategy tries to mitigate the impact or limitations that individual descriptors may have, by the collective evaluation of several descriptors to dilute potential shortcomings.

Based on the previous MMGBSA estimation, a more rigorous RT analysis is performed to evaluate the time in which the ligand remains stably bound, thus stabilizing the complex through preserved interactions over the simulation. Therefore, since ligand binding fluctuations may occur, ligand trajectories presenting free binding energy values exceeding the scaled standard deviation of its mean value, computed every 10 ns of simulation, are also discarded from the analysis protocol. To observe ligand stability and behavior, the time evolution of the computed RT is plotted at 5 Å and 25 Å *Y*-axis distance limit for each selected individual trajectory.

Moreover, solvent exposure plays a crucial role in terms of binding stability. Less solvent exposed ligands will translate into solvent-inaccessible deep hydrophobic cavities, with stronger stabilizing interactions, and thus favoring better affinities as an entropy response upon ligand binding. Hence, the assessment of the ligand exposed surface area accessible to the solvent (water) provides complementary information to comprehend ligand binding affinities. SASA calculations were performed using the “molsurf” algorithm, based on the Connolly method, as implemented in Amber22 CppTraj.^47,69^

Hence, to support decision-making in the final evaluation of protein–ligand individual trajectories, analysis of the hydrogen bonds has been chosen as the last and additional descriptor, to provide an integrated strategy to capture both energetics and structure, by contributing to a more reliable understanding of ligand–protein binding behavior. Unlike hydrophobic interactions or vdW forces, hydrogen bonds are directional and specific. Typically, these noncovalent interactions help the complex stabilize, effectively orienting the fragment in the correct binding mode. Consequently, donor–acceptor hydrogen bond interactions established during the last 20 ns of the trajectory are evaluated. Contributions greater than 45% of the evaluated time are considered in the final analysis. This additional descriptor is aimed at complementing the information extracted from the trajectories, considering the presence of hydrogen bond interactions as a good indicator for the selection process. Hydrogen bonds are computed using the CppTraj module from AmberTools.^47^

Overall, the use of multiple independent approaches to evaluate protein–ligand complex stability and binding affinity can strengthen confidence in the selection of the best binding site (and mode of interactions) by supporting the obtained results, and consequently the robustness of the methodology.

### Trajectory & Binding Analysis

After the production step, the 4 independent fdGaMD simulation replicas performed are stripped. By doing so, each fdGaMD trajectory containing multiple copies of the same fragment is stripped into individual ligand–receptor trajectories to be analyzed. Water molecules and counterions are eliminated at this stage to simplify the analysis and minimize computational time. Then, once obtained, each of the fragment trajectories is analyzed in the search for potential druggable binding sites.

To begin with the analysis, the minimum distance between the central atom of the studied fragment (**C**99/**N**99/**O**99/**S**99) and any atom of the protein is calculated at the end of each individual trajectory from all the independent fdGaMD simulation replicates performed. Then, fragments at distances greater than 5 Å of the target protein are discarded. Hereafter, an initial estimation of the RT based on the same distance criterion is done, only for ligands exhibiting protein–ligand interactions. Considering possible ligand fluctuations within the binding site along the trajectory, we define the descriptor as the simulation time in which protein–ligand distances are maintained under the prestablished interaction threshold for at least a 90% of the interaction time. Accordingly, as a preliminary screening step, only trajectories fulfilling the interaction criterion for at least the last RT cutoff of simulation are subsequently selected for the fdGaMD analysis, significantly reducing the computational cost associated. To determine the RT cutoff, we performed several tests and ultimately selected 50 ns because, together with the rest of descriptors, it was able to discriminate between good and bad binding sites/modes in all the systems studied. Then, the remaining descriptors are computed, as described in the previous section, for the so-called “reactive trajectories” by Privat et al.^31^ Representative results obtained from the fdGaMD analysis protocol can be found in Table S22.

Once obtained, descriptors are assigned to their corresponding binding site, processed and ranked considering contributions of all replicates (e.g., Tables S2–S21). As a result, we will end up with a binding site ranking consisting of the number of reactive trajectories for each site identified (being the number of replicates performed, the maximum value), best RT of the binding, as well as the best and averaged values for the *E*_inter_ and MMGBSA estimations, obtained during their respective RT for each binding site and the last 20 ns of simulation, the % of ligand exposed SASA computed at the last snapshot of the simulation and RT, as well as the highest number of hydrogen bonds involved in the ligand–receptor interaction for that specific binding site. Averaged values are computed for each descriptor as the summatory of the reactive trajectory values divided by the number of independent simulation replicates. These values are then analyzed to account for the number of best descriptor values per site (ranking). Finally, the binding mode presenting the best interactions and behavior for the most favorable binding site identified will be selected as the final predicted binding conformation for the system.

Since the maximum possible number of reactive trajectories for a given binding site corresponds to the number of independent runs performed, obtaining an elevated number represents a good indicator to show the probability of how feasible it is for the fragment to find and favorably bind onto that specific spot.

Cavities are characterized by different types of interactions: electrostatic interactions, hydrogen bonds, aromatic interactions such as π–π stacking, hydrophobic contacts and van der Waals interactions. Every binding site is unique as its environment, defined by a set of amino acids, makes it specific for a ligand to bind. To properly describe the previously selected sites, protein residues with atoms at distances lower than 4.5 Å to the ligand are identified and defined in lists. Aimed to distinguish between sites, similarities are evaluated by means of the Jaccard index (eq 16). To assign and classify the binding site as a new or a previously identified one, differences between sets of amino acids (A and B, for example, defining both binding sites to be compared) are quantified. All possible similarities between all the binding sites identified are evaluated. In our case, only those exhibiting a Jaccard index of J>0.35 are assigned as existent ones.

16 J(A,B)=|A∩B|/|A∪B|

Regarding stabilization criteria, if a fragment shows interesting descriptor values that stand out from the rest, but the time evolution of the descriptors fluctuates or shows rapid changes, it may indicate the nonconvergence of the system. In such cases, it is recommended to extend the simulation length and repeat the selection process in the search for more robust results. Hence, only those fragments clearly exhibiting the best descriptor values and behavior are selected for optimization purposes and drug development. Thus, a visual inspection of the most favorable binding mode of the selected binding site must be performed. The cavity is then explored in depth with the aim to grow the fragment into a larger drug candidate, focusing on increasing the number of interactions to stabilize the complex and increase its affinity.

Given that the selection process protocol derived from the analysis focuses on those ligands exhibiting the best descriptor values and behavior, it might occur that ligands with very weak interactions may not exhibit good affinities nor even be detected with the fdGaMD protocol.

## Results and Discussion

Following the general idea of the conventional fdMD approach,^31^ GaMD simulations have been performed instead, with the aim of improving binding site prediction and binding mode elucidation. Moreover, changes such as the principal axes ligand orientation to efficiently build the simulation boxes, the optimization of the GaMD protocol, and variations in the descriptors chosen for each individual trajectory analysis have also been implemented for improvement. The fdGaMD approach has been validated using 12 different biomolecular ligand–receptor complexes classified in three different data sets for benchmarking, acceleration effect evaluation, and exploration of its limitations (refer to Table S1 and “Validation: Selection of the Studied Systems” subsection of Computational Methods). For each system, 4 independent fdGaMD simulations of 400 ns each have been performed, analyzed, and compared with experimental data. Details regarding the protocols for preparation, production, and trajectory analysis can be found in the Computational Methods section of the manuscript. Results are discussed in detail below.

### Set I: Conventional fdMD Methodology as a Benchmark

To assess the effectiveness of introducing GaMD simulations within the conventional fdMD approach, it is essential to first establish a benchmark to compare the performance of the improved methodology against the original one. Aimed to reproduce one of the most typical FBDD scenarios in which the binding mode is unknown, one must consider the target protein structure in its free-state unbounded conformation when applying the methodology, to account for both conformational selection and induced fit. For this purpose, we selected the apo structure of the myeloid cell leukemia 1 (MCL-1) protein,^53^ known to experimentally bind to 3-chlorobenzo­[*b*]­thiophene-2-carboxylate (class I) and 3-(4-chloro-3,5-dimethylphenoxy)­propanoate (class II) fragments with an inhibition constant (*K*_i_) of 131 μM and 60 μM, respectively.^52^ In this case, despite having experimental affinities, the only crystallographic structure available was the one corresponding to a larger ligand, with PDB ID 4HW3, obtained by linking both active fragments (2D representation of the structures is available as Figure S1).^52^

In concordance with the proposed fdGaMD protocol and the validation of the conventional fdMD approach,^31^ 4 independent 400 ns fdGaMD simulations were performed for both fragments (class I & II). Therefore, binding poses were assessed after 200 ns of simulation, despite performing the complete simulation length analysis protocol, to ensure direct comparability with the conventional methodology. The results (Figure 4 and Tables S2 and S3) demonstrated a significant improvement using the alternative fdGaMD approach. In contrast to the conventional methodology, which was limited to identifying the most favorable binding site, the accelerated fdGaMD simulations accurately captured the native binding mode and interactions of fragment class I in its preferred site (Figure 4A). Thus, 200 ns fdGaMD were sufficient to correctly reproduce the crystallographic orientation of the ligand through a stabilizing directional hydrogen bond interaction between the carboxylic group of the ligand and protein arginine 263 (R263). In fact, residue R263 has been reported to play a crucial role in ligand binding, typically through hydrogen bonding or electrostatic interactions, at the orthosteric site of the BCL-2 protein family.^70–72^ The residue—highly conserved in the BH3-binding groove—can form salt bridges with aspartic acid 256 (D256), stabilizing its structure, or interact with BH3-only proteins and peptides (representing the BH3-Noxa or BH3-Bim domain) through a conserved aspartic acid.^73–75^ In this context, carboxylic groups can act as ideal acceptors of R263 interactions and are commonly found in BCL-2 protein family inhibitors, such as navitoclax (ABT-263) or AbbVie’s inhibitor A-1210477 and its derivatives, which disrupt stabilizing R263-D′ salt bridges by BH3 mimetics.^76–78^

**Figure 4:**
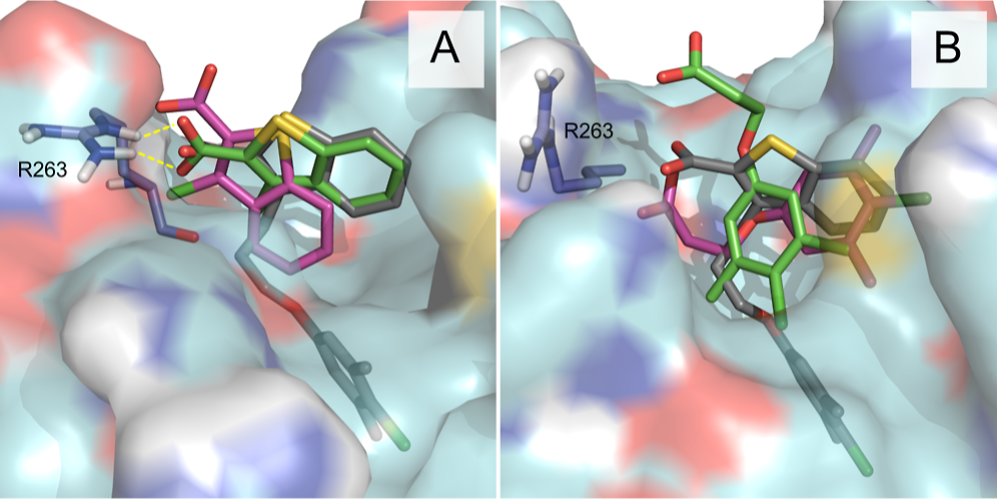
Representation of the most favorable binding modes identified for fragments “class I” (A) and “class II” (B) of MCL-1 system, validation set-I. Predicted fragment poses obtained after 200 ns of conventional fdMD (magenta) and accelerated fdGaMD (green) are depicted and superposed to the X-ray crystallographic structure of the larger ligand (gray), with PDB ID 4HW3. The simulated protein surface and key interacting protein residues are shown in cyan and deep-blue colors, respectively. Stabilization of the carboxylic group of the ligand, by means of hydrogen bond formation (yellow dashed lines) with key protein residue R263 is also represented. Nonpolar hydrogen atoms are omitted in both figures for clarity.

In contrast, the class II fragment was more challenging to reproduce. The fragment appeared to adopt better carboxylic group and aromatic ring orientation, despite possible conformational restraints that may have hindered its deeper insertion into the binding pocket. Since no crystallographic structures are available for the individual fragments, we are limited in providing a definitive interpretation of the results. Even though, the improved pose and the high affinity of the interactions stablished between the carboxylic group and R263 suggest better ligand accommodation compared to the fdMD approach (Figure 4B). Moreover, nuclear Overhauser effect (NOE)-guided fragment docking and nuclear magnetic resonance spectroscopy (NMR)-derived models have shown class I and class II to selectively bind to the orthosteric binding pocket, with the carboxylic acids of both fragments pointing toward R263, consequently confirming their competitive nature that prevented both compounds from simultaneously binding experimentally to MCL-1.^42^ Notably, these reported results align with the fdGaMD predicted ligand orientation and binding mode obtained for both fragments, especially elucidating class II pose.^52^

Overall, the fdGaMD approach demonstrated significant advantages over the conventional methodology, specifically given the importance of accurately predicting native interactions for fragment-based drug design and subsequent ligand optimization. The acceleration introduction enabled a better conformational sampling and boosted convergence toward adaptation into the native binding mode of the ligand. This enhanced sampling efficiency therefore allowed the ligand to adopt favorable interactions within the binding site, highlighting the ability of the improved methodology to predict and identify the most favorable binding site as well as elucidate its native interactions and mode of binding.

### Set II: Affinity Matters

Accelerating the exploration of the conformational space of the target protein can enhance binding site identification and binding mode elucidation, as previously discussed. Ligands exhibiting strong native interactions toward the target protein corresponding to favorable and well-preserved local minima states, will result less prone to being affected by the acceleration boost introduced. However, due to the smoothing of the energy barriers, weak fragments may present less stability when applying the acceleration boost, while easing the exploration of sometimes spurious, more accessible sites. In this context, validation set-II was tailored to evaluate the effect of the acceleration in terms of ligand affinity. For this purpose, three fragments known to bind to the urokinase-type plasminogen activator (uPA) protein, with available crystallographic structures (PDB ID 1F5K, 4FU8 and 4FUD) and increasing experimental binding affinities (*K*_i_ of 100 μM, 6 μM and 0.5 μM, respectively), were selected (Table S1).^54^

For the first and smaller fragment (BEN; PDB ID 1F5K), exhibiting weaker experimental affinities,^54^ both fdMD and fdGaMD approaches explored the ligand native interactions toward the protein, stabilized by means of hydrogen bond formation between aspartic acid protein residue D192 and the charged amine groups of the fragment, as can be observed in Figure 5A. While the experimental binding mode was identified as the most favorable for the fdGaMD approach (Table S4), the conventional fdMD method failed at the identification of the native interactions, although being explored at the end of the simulation (Figure 5A, Table S5). However, an in-deep analysis of the results revealed that relaxing the threshold applied for trajectory selection made possible the detection of the experimental binding site, but not the recognition of the native interactions as the most favorable for the system (Table S6). Since small organic molecules often show to be more promiscuous in diverse binding toward the protein, it is not rare to observe this type of behavior as a consequence of their known lack of selectivity (Figure S2), established as one of the main challenges in FBDD.^79^ Fortunately, the accelerated fdGaMD method was able to identify the experimental binding site as the most favorable from the analysis (Table S4). Nevertheless, the enhanced binding site exploration—enabled by the acceleration applied—helped to discard spurious results. However, weak ligand interactions led to reduced RT values, which posed a challenge to the protocol.

**Figure 5:**
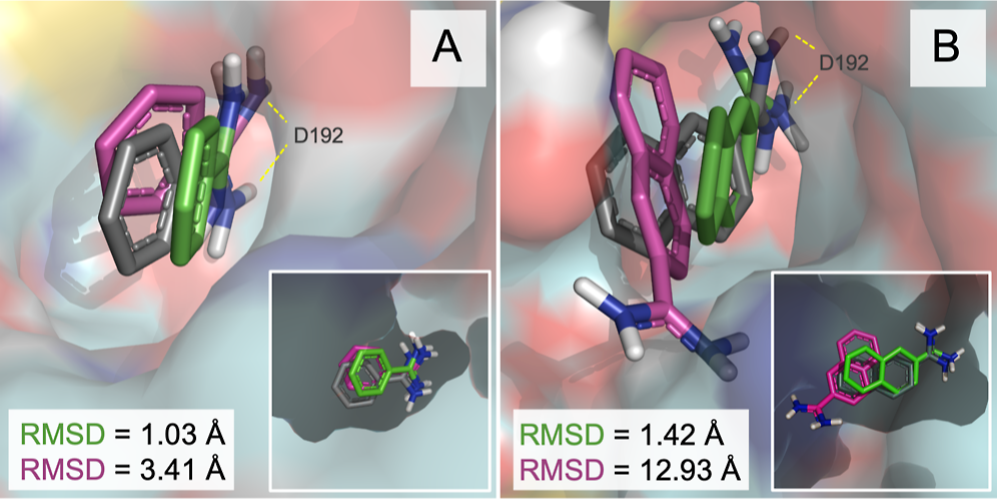
Representation of the binding modes identified for fragments BEN (A) and 2UP (B) of urokinase uPA systems with respective PDB ID codes 1F5K and 4FU8, from validation set-II, and bottom-right depiction of the ligands at the orthosteric pocket cavity. Predicted fragment poses obtained after 400 ns of accelerated fdGaMD (green), and 400 ns (A) and 375 ns (B) of conventional fdMD (magenta) are depicted and superposed to the X-ray crystallographic structure of the complexed ligand (gray), with the simulated protein surface shown in cyan. Illustration of the hydrogen bond formation with key protein aspartic acid residue D192 as yellow dashed lines. Nonpolar hydrogen atoms are omitted in both figures for clarity. RMSD values corresponding to the last 100 ps of the complete simulation are shown; detailed information is available in Table S32.

Regarding the intermediate size fragment (2UP; PDB ID 4FU8),^54^ the introduction of acceleration into the main methodology resulted in a correct capture of native key interactions leading to the elucidation of the experimental mode of binding (Figure 5B), outstanding as the most favorable site identified from the final analysis performed (Table S7). In contrast with the results obtained with the fdGaMD methodology, no ligand was found bound to the experimental binding site at the end of the conventional fdMD simulations performed (Table S8). Closer inspection of the trajectories showed that the experimental binding site was indeed visited once during the simulation, although with an unfavorable orientation that subsequently led to final ligand unbinding after 375 ns of fdMD simulation (Figures 5B and S3). Without enough sampling to allow the fragment to favorably visit the pocket and permit proper protein accommodation for stablishing stable native interactions, the conventional fdMD approach demonstrates limitations in exploring and correctly reproducing the experimental binding conformation. Hence, for ligands exhibiting moderate binding affinities, fdGaMD offers a clear advantage in binding mode elucidation due to its enhanced sampling efficiency.

The last and larger fragment (6UP; PDB ID 4FUD),^54^ presenting the best experimental affinity in our validation set-II, led to the identification of the experimental binding site from both conventional and accelerated simulations (Figure 6A).

**Figure 6:**
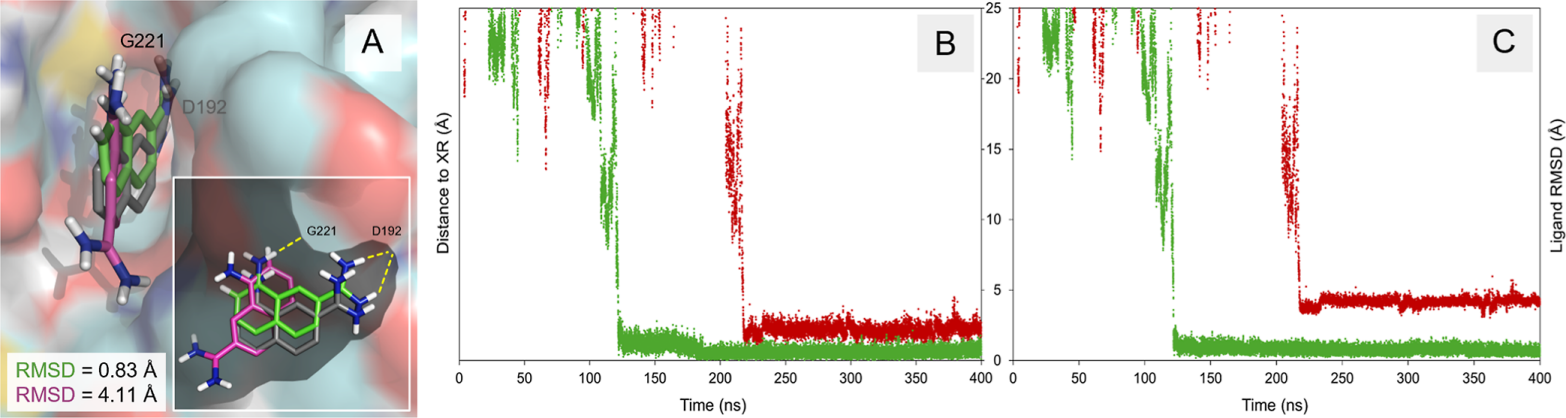
Representation of the most favorable binding mode identified for ligand 6UP (urokinase system uro0003; with PDB ID 4FUD) from validation set-II, with predicted fragment poses obtained after 400 ns of conventional fdMD (magenta) and accelerated fdGaMD (green) simulations are depicted and superposed to the X-ray (XR) crystallographic structure (gray), with the simulated protein surface shown in cyan (A). Bottom-right depiction of the ligands at the orthosteric pocket cavity, with hydrogen bonds formed with key protein aspartic acid residue D192 and glycine G221 represented as yellow dashed lines. Nonpolar hydrogen atoms are omitted in the figures for clarity. Distance of the simulated ligand with respect to its crystallographic conformation (B) and ligand RMSD (C) profiles, obtained for the fragment interacting at the most favorable binding site identified over the 400 ns of conventional fdMD (red) and accelerated fdGaMD (green) simulations performed. The crystallographic X-ray (XR) structure of the system has been employed as reference for both analyses. RMSD values corresponding to the last 100 ps of the complete simulation are provided in Table S32.

Again, notably, only the fdGaMD approach correctly reproduced the native binding mode—involving aspartic acid D192 and the carboxyl backbone group of glycine G221 key protein residues—thus standing out as the most favorable binding site from the analysis (Tables S9 and S10). Moreover, accelerating the conventional fdMD approach enabled a faster and more efficient exploration of the experimental site (Figure 6B), even before the first 200 ns of simulation. Not only did the fragment arrive faster, but it also improved its conformation compared to the X-ray structure, as depicted by the ligand root-mean squared deviation (RMSD) shown in Figure 6C. As a strong binder, cMD simulations may trap the fragment in relatively stable local minima corresponding to spurious conformations, hindering the unbinding events within the range of typical simulation time scales. Consequently, conventional methods may be less effective at capturing and characterizing specific interactions between the fragment and the explored target protein, thus highlighting a critical aspect in the early stages of computational FBDD: to enable proper binding mode elucidation of the “hit” fragment compound to rationally design the further optimized and larger “lead” drug candidate.

Aligned with the initial hypothesis, the results presented for validation set-II have demonstrated the advantages of accelerating fdMD, especially for moderate and strong binders. The introduction of the GaMD potential boost, added to smooth the potential energy surface of the system, has significantly improved conformational sampling and consequently favored the identification of binding sites and binding mode elucidation. From a ligand affinity perspective, fdGaMD can provide significant advantages in the FBDD field, especially in scenarios where cMD approaches are limited by insufficient sampling. With increased binding probability due to the use of multiple fragment copies in the simulation and the enhanced system exploration provided by the acceleration boost,^31,33^ fdGaMD has demonstrated to be a powerful tool for binding mode elucidation, thus presenting a valuable impact on rational drug design.

### Set III: Exploring the Limitations of fdGaMD

Proceeding with the fdGaMD validation, the last set—consisting of 5 diverse complexes with experimental binding affinities in the range from unavailable data to values from 10^3^ to 10^–1^ μM—was selected to explore the limitations of the presented methodology (as described in Computational Methods “Validation: Selection of the Studied Systems” subsection). We expected, therefore, the results to be more straightforward and conclusive in the case of fragments showing a higher ability to form interactions and presenting higher experimental affinity than, on the contrary, fragments in low-affinity scenarios that could potentially result in their dismissal.

In the absence of experimental affinity assessments for system 1I06, we initially assumed the small TZL fragment to exhibit weak affinities toward its target, the major urinary protein I (MUP-I).^61^ To assess the robustness of the method in such cases, we started by using the standard fdGaMD protocol (as described in Computational Methods). Our preliminary analysis, using the standard RT cutoff of 50 ns, was inconclusive since only a single binding site, not corresponding with the experimental one, was detected exhibiting estimated binding energies around −10 kcal/mol, a relatively low value compared to previous systems from sets I and II (Table S11). Aimed to determine the limitations of the approach, we performed a second analysis using a less restrictive RT cutoff of 20 ns to increase the number of trajectories that satisfied the RT cutoff criteria to continue with the subsequent steps of the analysis. By doing so, it was possible to identify and select the most favorable binding site for the fragment, exhibiting estimated binding energies around −20 kcal/mol, twice as favorable as the spurious site previously identified (Table S12 and Figure 7A). However, in such short residence times, the specific experimental binding mode could not be correctly elucidated due to the absence of directional interactions which typically favor proper ligand orientation and positioning. Hence, the acceleration introduced may challenge the elucidation of fragments exhibiting low RT because of their inherent weak interactions. These weak fragments typically remain bound only brief intervals of time, occasionally exhibiting binding and unbinding events (see Figure S4), leading to their dismissal by the protocol. From a virtual screening point of view, this can be beneficial for effortlessly discarding low-affinity compounds thanks to the introduction of GaMD. Nevertheless, when seeking to determine the best binding site in low-affinity scenarios, cutoff parameters can be flexibly adjusted to accurately capture more binding events in the search for druggable hotspots, as demonstrated with system 1I06.

**Figure 7:**
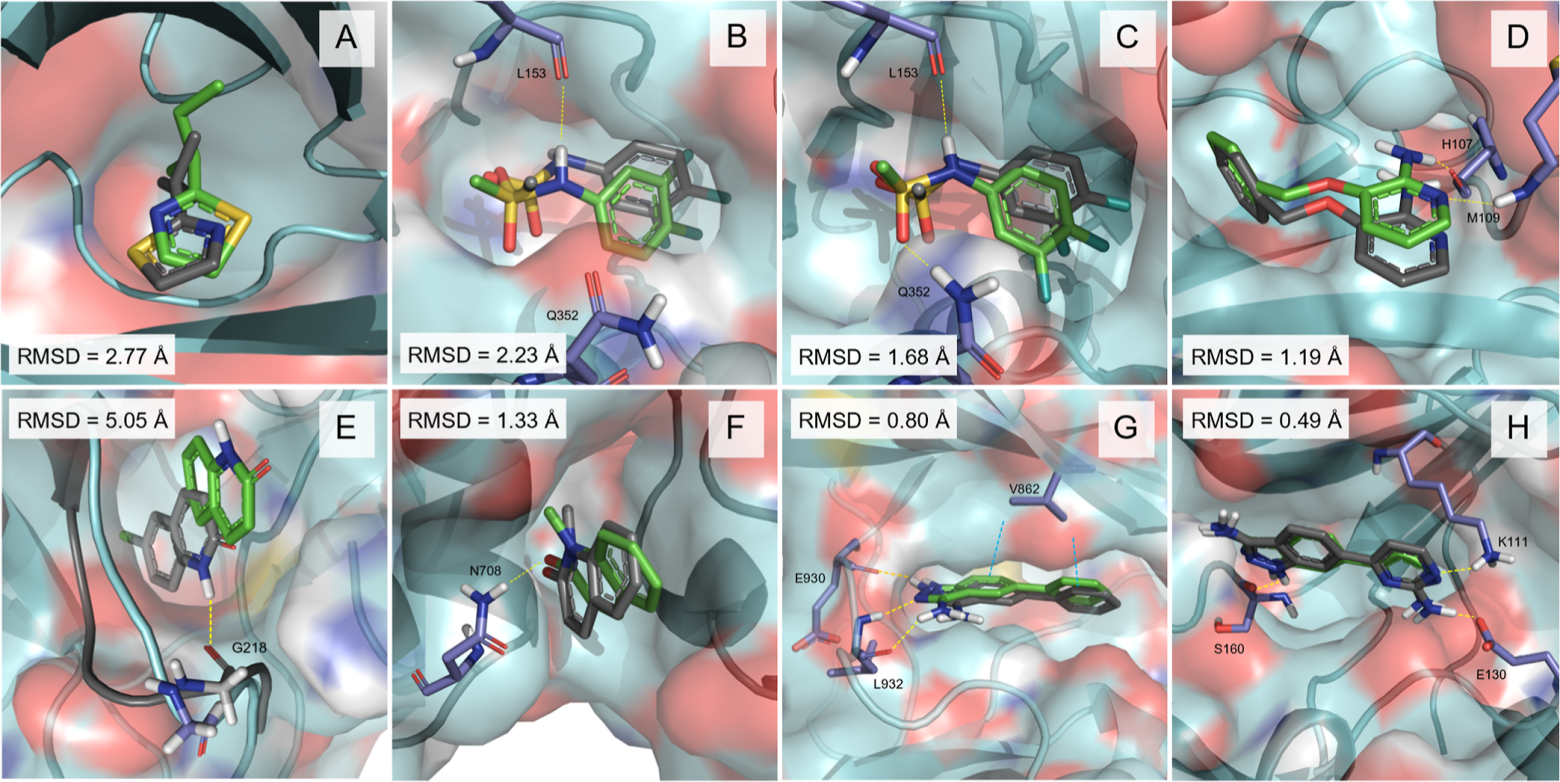
Predicted binding poses after 400 ns of fdGaMD simulations (green) for “set-III” of diverse biomolecular ligand–receptor systems superposed to their respective X-ray crystallographic structures (gray), with PDB ID 1I06 (A), 5YE8 (B,C), 1W7H (D), 4CR5 (E), 5T4U (F), 3E63 (G) and 3NUN (H). Simulated target protein structure (surface and cartoon) represented in cyan, with key binding site amino acids depicted in deep blue. Hydrogen bonds and hydrogen–arene interactions are respectively represented as yellow (B–H) and blue dashed lines (G). Nonpolar hydrogen atoms are omitted for clarity. RMSD values corresponding to the last 100 ps of the complete simulation are shown; detailed information is available in Table S32.

Proceeding with the protocol validation and the performance assessment for fragments in a low-affinity range, system 5YE8 was selected for the study as it presents an experimental binding affinity of 10^3^ μM.^55^ The analysis made it possible to correctly identify the experimental binding site as the most favorable by the fdGaMD protocol (Table S13). However, the rotational flexibility of the 8UT fragment 3,4-difluorophenyl group challenged the elucidation of the most favorable binding mode for the complex (Figure 7B). In the PDB crystallographic structure, the ligand is stabilized at the pocket through a hydrogen bond interaction between its amino group and the backbone carbonyl group of leucine L153 residue, along with water-bridged hydrogen bonds established with residue neighbors.^55^ Then, through stabilization of the 8UT sulfonamide tail, interactions at the binding site were maintained during the simulations but, due to the hydrophobicity of the pocket region—defined by protein residues L107, F110, G154, A155, and L159—no clear interaction directionality for the 3,4-difluorophenyl group was observed.^55^ As shown in a short Supporting Information Video (generated with UCSF Chimera software),^80^ rapid conformational changes are prone to occur probably due to the energetic barrier associated with the sulfonamide-aromatic N­(sp^2^)–C­(sp^2^) bond rotation, assumably lower than for typical benzamidines as a result of the decreased conjugation and weaker resonance stabilization of sulfonamides.^81^ Then, although two conformations were identified along the trajectory indistinctly (Figure 7B,C), the last snapshot of the simulation revealed the rotated binding mode depicted in Figure 7B.

Similarly, the 1W7H system with an experimentally determined binding affinity in the range of 10^2^ to 10^3^ μM,^56^ was included for validation. After conducting the fdGaMD protocol, the experimental binding mode stood out as the most favorable across all the descriptors applied (−31 kcal/mol; Table S14 and Figure 7D). Notably, system 1W7H is a good example of a slightly larger ligand fragment that can form stabilizing interactions, thus facilitating the ligand to adopt its conformation within the binding site. Typically, the larger the ligands are, the greater their capacity to interact with protein residues. In this case, hydrogen bonds with the backbone of residues M109 and H107, along with stabilizing hydrophobic interactions between the ligand benzene substituent and surrounding residues, contributed to the overall stabilization of the complex binding conformation.

For system 4CR5,^57^ on the contrary, the approach was not sufficient to identify the most favorable interactions as the ones corresponding to the experimental binding site, despite exhibiting a good energetic profile (Table S15 and Figure S5). Analysis of the results revealed that fragment 0UT binds to the experimental site in a prepocket state, adopting an orientation inverted relative to the crystallographic pose (Figure 7E). Being observed in just one of four replicates and in an orientation incompatible with favorable interactions, this suggests that sampling was inadequate to capture the native binding conformation, as discussed in detail in the following section.

On the other hand, as observed in Figure 4F–H, the same protocol performed for systems 5T4U,^58^ 3E63,^59^ and 3NUN^60^—with corresponding experimental binding affinities of 4.8 μM, 1.6 μM, and 0.37 μM—proved to accurately reproduce the experimental binding mode of their respective fragments 12Q (Figure 7F), 5B2 (Figure 7G) and JMZ (Figure 7H), with only 400 ns of fdGaMD simulation (Tables S16–S18). All three systems made evident that strong and directional interactions play a key role in the stabilization of the complex, thus favoring its adaptation toward the native orientation of the ligand within the binding site. Therefore, this highlights the importance of favorable molecular interactions, not only as a metric of favorable binding affinities, but also as a means to enable an accurate binding mode elucidation. Our results (Tables S1 and S2–S18) further corroborate this consistent relation between experimental binding affinity and the specificity of protein–ligand interactions, which directly translate into a clear and unequivocal mode of binding. Overall, the integration of GaMD simulations into the original fdMD protocol provides a robust and promising strategy for effective binding mode elucidation, particularly for ligands with affinities below the micromolar range.

Finally, the analysis of the last validation set enabled a comprehensive identification of the limits and potential of the fdGaMD protocol across a wide range of experimental binding affinities. Using the fdGaMD analysis protocol descriptors, we observed that the more favorable, stable, and specific pocket interactions led to accurately elucidated binding poses. Our findings confirmed the robustness of the protocol in capturing and reproducing the correct native interactions and subsequent binding conformations, particularly for systems presenting higher experimental affinities (such as 5T4U, 3E63, and 3NUN). Considering the consistency between predicted and experimental poses for high-affinity ligands, as well as the favorable dismissal trend for flexible or low-affinity binders, the fdGaMD methodology can potentially help in the early stages of fragment-based drug design studies. For moderate- to high-affinity fragment–protein complexes, fdGaMD can reliably identify the most favorable site and mode, thereby facilitating hit-to-lead optimization protocols. While on the contrary for weak and flexible fragments, or very water-exposed binding modes, the fdGaMD approach can help effectively discern and discard those fragments during screening protocols.

### Going Further the Standard fdGaMD Protocol: The Crucial Role of Ligand Trajectories in Binding Mode Elucidation

As discussed in the previous section, the standard approach was unsatisfactory to identify the experimental binding site of the 4CR5 system as the most favorable. We observed that, even if a binding site is visited, the fragment must show the proper interactions to stabilize the complex. Hence, if the ligand arrives in an orientation different from the native one, descriptors will not point toward it since the interactions between the fragment and the target protein will not be as favorable as the corresponding for the experimental binding mode.

All this considered, the experience led us to question whether the simulation time was insufficient for both the ligand to arrive with a favorable orientation and the protein to facilitate the conformational adaptation of the complex. Furthermore, we also questioned not only the relevance of the time needed to stabilize and find the most affine binding mode but also the impact of the probability of entering the pocket in the proper orientation.

Going beyond the general scheme of the fdGaMD methodology, we extended the simulation time to 1 μs for each of the four independent replicates performed. As anticipated, the fragment preserved its initial binding orientation throughout the simulation. Over the additional 600 ns of simulation performed, no significant conformational change occurred for the fragment, aside from a brief, transient period of destabilization from which the ligand quickly recovered to adopt the same initial orientation (Figure S6). No further binding events were observed during the extension protocol, leading us to assume it may be related to a probabilistic issue. Consequently, we performed 12 new independent fdGaMD replicates of 400 ns each to corroborate our hypothesis.

The results showed that, with enough data, the experimental binding mode could be explored and identified as the most favorable binding site and conformation (Table S19). In 4 of the 12 replicates, the ligand was found interacting at the crystallographic site but adopting different binding modes, reflecting its relatively low binding affinity (140 μM; Table S1 and Figure 8). Even so, despite the subtle differences between conformations (Table S20), the fdGaMD approach was able to discern the experimental binding mode as the most favorable of the ones identified (native mode, Figure 8A). While the native mode was once identified, two different replicates exhibited a rotated conformation (from now on flipped mode, Figure 8B), thus deviating from the ideal binding pose. In addition, the last fragment was identified at the binding site with a fully backward orientation, as can be appreciated in Figure 8C. Interestingly, we found fragments presenting different binding modes in a metastable state near the experimental binding pose, each of them showing different RMSD fluctuation profiles (Figure S7).

**Figure 8:**
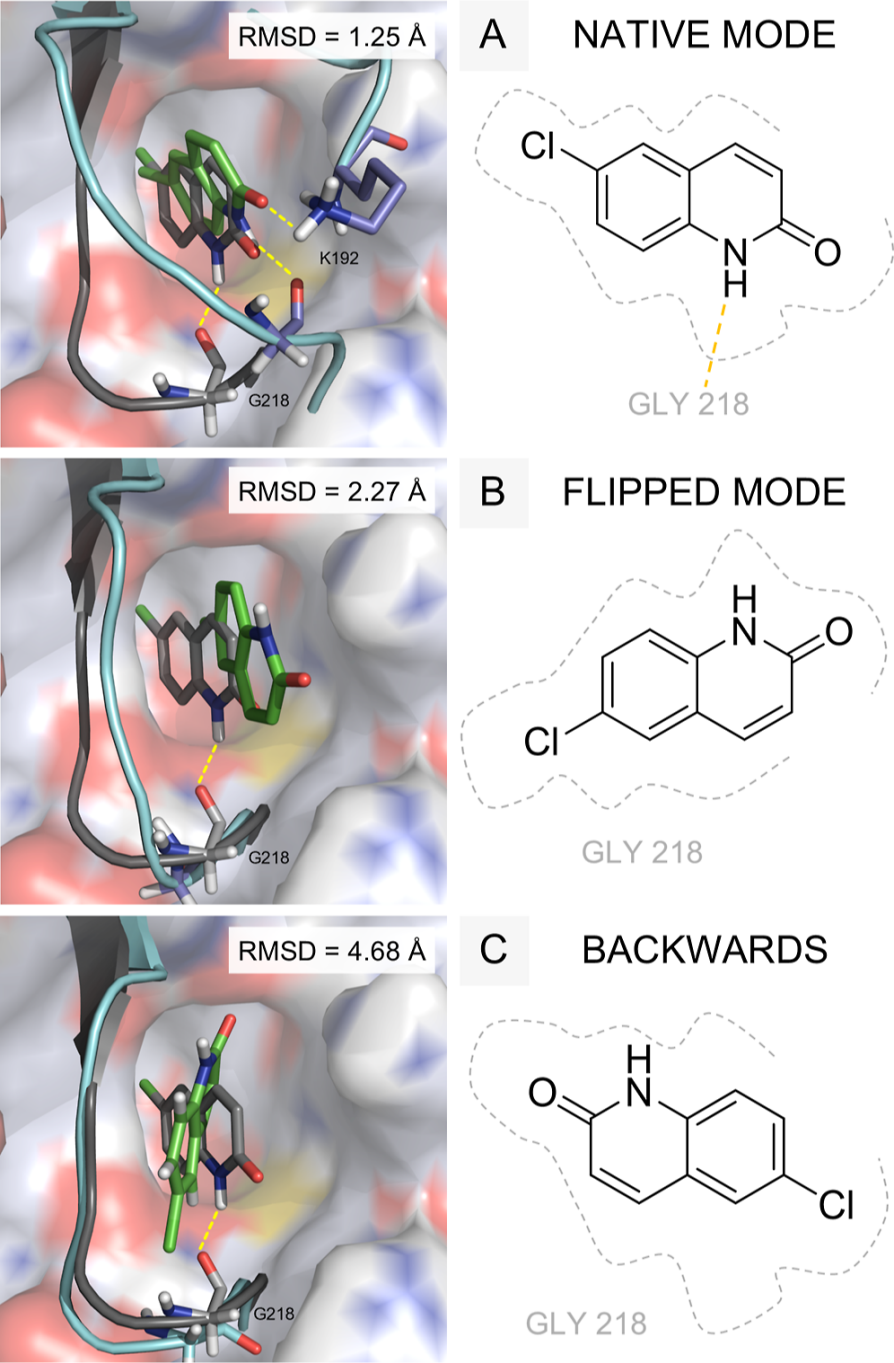
Representation of the different binding modes identified for system 4CR5 after performing 12 fdGaMD independent replicates of 400 ns. From left to right, the native (A), flipped (B), and backward (C) conformations are shown in both 2D and 3D representations. Each panel displays the experimental binding pose (in gray) alongside the predicted pose (in green) obtained after 400 ns of simulation, superimposed on the crystallographic structure (also in gray). Protein surface, loop and relevant amino acids are depicted as cartoon and sticks, in gray or blue tones for the X-ray or simulated structures, respectively. Nonpolar hydrogen atoms have been omitted in the figures for clarity. As appreciated, protein residues G218 and K192 showed to play a crucial role to adopt the native mode of binding due to a stabilizing hydrogen bond interaction, represented as yellow dashed lines. RMSD values corresponding to the last 100 ps of the complete simulation are shown; detailed information is available in Table S32.

Without the realization of a more extensive study based on multiple replicates, binding mode elucidation would not have been possible unless the ligand showed the proper orientation. Consequently, the results obtained led us to question: is it then a statistical problem only? To evaluate the premise, 48 new independent fdGaMD replicates of 100 ns each were performed to be comparable with previous results (Table S21). The trajectory analysis revealed that the binding site corresponding to the experimental data was identified with the proper orientation, in a prepocket state, but not exhibiting the experimental binding mode (Figure S8). Additionally, it is interesting to highlight that—independently of the orientation—all the ligands identified at the experimental binding site were found in a prepocket binding state, similarly to the one identified in the validation data set simulations, with a RT of 110 ns (Figure 7E and Table S15). These results suggest that short simulation times may not be sufficient to allow the receptor to adapt and correctly receive the ligand in its proper orientation, to establish native interactions. Consequently, it is important to not only perform enough replicates to capture conformational selection, but also to provide enough time for the ligand to accommodate into the cavity and for the complex to adapt (induced fit).

Consistent with the work of Shaw et al.,^82–84^ achieving the native binding pose requires the ligand to traverse a pathway of favorable metastable intermediates or prebinding conformations. Having enough time to explore the protein and let the protein adapt to the new complexed conformation is essential, especially for cases unraveling potentially cryptic binding sites. Accordingly, our results suggest that approaches based on the use of multiple short simulations will be limited at capturing the native interactions of more complex systems, as the ones presented in this work.^85^ Achieving accurate binding predictions requires more than probabilistic sampling, as often relies on conformational exploration toward native conformation adaptation. Statistically, arriving with the proper orientation to a binding site will facilitate the ligand to adapt and finally reach the most stable and favorable interactions (as sometimes it is challenging to escape local minima) with enough simulation time. Therefore, increasing the amount of data can improve the identification of the most favorable binding site by enhancing protein exploration. However, accurately reproducing the experimental binding mode requires sufficient simulation time for the ligand to adopt its native conformation. This highlights the importance of considering not only energetic favorability but also the directionality and specificity of molecular interactions.

Beyond the initial fdGaMD strategy (4 × 400 ns), more extensive and varied protocols can be implemented to further refine the results, especially in more complex or ambiguous systems. Ideally, increasing the number of independent replicates performed along with extended simulation lengths enhances the robustness of the obtained results. However, since challenges reproducing the correct binding mode are often associated with low-affinity compounds, it does not represent a substantial limitation for potential massive fdGaMD studies. On the contrary, it can be beneficial, as fragments with unclear, unstable interactions are more prone to be disregarded in the first steps of the analysis, thus easing the protocol. Therefore, we will end up with only the best fragments in their most favorable binding site and mode. Consequently, the introduction of GaMD to the fdMD approach can significantly simplify screening processes, focusing on the most promising and properly elucidated fragments.

## Conclusions

To overcome the limitations of conventional molecular dynamics (cMD) in the fragment dissolved molecular dynamics (fdMD) approach,^31^ we propose the alternative use of Gaussian accelerated molecular dynamics (GaMD).^33^ Smoothing the energy landscape can facilitate the exploration of conformational states that otherwise would be challenging to sample within standard MD simulation time scales. Hence, by applying a boost potential to the energetic profile of the system, the fragment dissolved Gaussian accelerated molecular dynamics (fdGaMD) methodology has permitted a faster and more efficient exploration of the conformational space with multiple copies of the same fragment simultaneously sampling the target molecule in a reasonable computational time of 400 ns of global simulation.

Regarding the identification of the most favorable binding site, fdGaMD has demonstrated to be capable of reproducing the experimental binding mode for the studied cases. Not only has accelerated binding site detection but also has facilitated the selection of the most affine interactions for each studied complex. Despite the significant increase in potential spurious hotspots identified due to enhanced sampling, the proposed descriptors have been sufficient to disregard and classify all the individual trajectories, clearly defining the most favorable one. Overall, the best binding site of a fragment undoubtedly corresponded to the one presenting the best descriptor values, standing out from the rest. Nevertheless, challenging scenarios in which any binding site is notably outstanding from the ones explored, especially for ligands presenting weak interactions, may difficult the final decision process. In such cases, the standard simulation length of the fdGaMD protocol (400 ns) or the number of replicates performed (4) may not be sufficient to capture the native conformation of the ligand. Consequently, for systems of interest under this scenario, we recommend going beyond the standard procedure to resolve the binding mode and favor the selection process.

From an application perspective, along with the present work, we observed that fragments presenting weaker interactions toward the target protein are neglected because of the potential boost introduced. This consideration is particularly relevant for future studies, where the large-scale application of fdGaMD as an additional step in virtual screening campaigns will facilitate the prioritization of fragments with stronger interactions. The exclusion of weak binders can thus be considered as a strategic advantage in the identification of potential drug candidates.

Furthermore, fdGaMD can lead to a promising strategy for the identification of novel potential allosteric inhibitors. Due to the enhanced conformational space exploration performed, we consider the approach as an attractive strategy for cryptic pocket revealing and allosteric binding site elucidation, especially in cases where the orthosteric site is not the most favorable one or even when interesting hotspots show promising affinities. Since fdGaMD is devoted to FBDD studies, fragments are conceived as starting points to be optimized into larger molecules to become potential drug candidates. Consequently, after validation of the approach, in the present work we proposed a method being capable of accelerating binding site detection in a relatively short simulation time and disregarding low affinity systems, due to the better ligand–receptor conformational space exploration performed. Thus, fdGaMD represents not only a significant improvement over the original fdMD strategy, but also a step forward in elucidating reliable structures of protein–fragment complexes.

Finally, it should be noted that we have observed unbinding events, but only for weak fragments that ultimately form unstable interactions and are discarded during analysis. However, we have not observed unbinding processes for ligands with good binding energy values once they have bound to the protein at the experimental binding site and established the correct interactions. This observation poses a challenge for future developments of the method. In this direction, a suitable approach might be to selectively accelerate the protein–ligand interactions.^86^

## Supplementary Material





## Data Availability

Predicted binding modes obtained from the last snapshots of the fdGaMD simulations, superposed with their respective crystallographic structures, and examples of the inputs used for molecular dynamics and analysis are publicly available at https://github.com/DrugDesignUBUJA/fdGaMD. Scripts implementing the fdMD approach can be accessed at https://github.com/DrugDesignUBUJA/fdMD. For fdGaMD, Amber22 and AmberTools software packages were employed for MD production and subsequent analyses and are freely available from the academic AMBER distribution (https://ambermd.org).

## Notes

Details of the selected validation systems (Table S1 and Figure S1), the results obtained from the individual trajectory analysis performed (Tables S2–S22 and Figures S2–S8) along with binding site assignation (Tables S23–S31), RMSD analysis of the last 100 ps of selected trajectories (Table S32), and computational details regarding principal axis orientation (Figure S9) (PDF)
Additionally, a representative segment of the ligand individual trajectory capturing 3,4-difluorophenyl ring rotation events of system with PDB ID 5YE8 (MP4)

## Abbreviations

AMBER: assisted model building with energy refinement
BCL-2: B-cell lymphoma 2
BH3: BCL-2 homology 3
BS: binding site
CADD: computer-aided drug design
cMD: conventional molecular dynamics
FBDD: fragment-based drug discovery
fdGaMD: fragment dissolved Gaussian accelerated molecular dynamics
fdMD: fragment dissolved molecular dynamics
FF14SB: AMBER force field 14SB
FXIa: activated coagulation factor XIa
GAFF2: general AMBER force field version 2
GaMD: Gaussian accelerated molecular dynamics
GB: generalized Born
HTS: high-throughput screening
JAK-2: tyrosine-protein Janus kinase 2
Lp-PLA2: platelet-activating factor acetylhydrolase
MAPK14: mitogen-activated protein kinase 14
MCL-1: myeloid cell leukemia 1
MD: molecular dynamics
MM: molecular mechanics
MMGBSA: molecular mechanics generalized-Born surface area
MUP-I: major urinary protein I
NOE: nuclear Overhauser effect
PDB: Protein Data Bank
PDK-1: α-phosphoinositide dependent kinase 1
RESP: restrained electrostatic potential
RMSD: root-mean-square deviation
RT: residence time
SA: surface area
SASA: solvent accessible surface area
SMD: steered molecular dynamics
TIP3P: transferable intermolecular potential with 3 points
uPA: urokinase-type plasminogen activator
vdW: van der Waals
XR: X-ray
