# Supplementary material for: Boosting Drug Discovery: Expanding the Applicability of Fragment Dissolved Molecular Dynamics to Accelerate Binding Mode Elucidation
Source: J Chem Inf Model. 2025 Nov 24;65(23):12879–93. doi: 10.1021/acs.jcim.5c02122 (PMC12690574; doi:10.1021/acs.jcim.5c02122)
Supplement: Supplementary file 1 [file ci5c02122_si_001.pdf]

SUPPORTING INFORMATION

Boosting Drug Discovery: Expanding the Applicability of Fragment Dissolved Molecular Dynamics to Accelerate Binding Mode Elucidation

Maria Nuria Peralta-Moreno,† José M. Granadino-Roldán,‡ Maria Santos Tomas,ǁ and Jaime Rubio-Martinez.*,†

† Departament de Ciència dels Materials i Química Física, Universitat de Barcelona (UB) and the Institut de Quimica Teorica i Computacional (IQTCUB), Martí i Franqués 1-11, 08028 Barcelona, Spain.

‡ Departamento de Química Física y Analítica. Universidad de Jaén, Campus "Las Lagunillas" s/n, 23071 Jaén, Spain.

ǁ Department of Architecture Technology, Universitat Politecnica de Catalunya, Av. Diagonal 649, 08028 Barcelona, Spain.

* Correspondence and requests for materials should be addressed to J.RM.

(email: jaime.rubio@ub.edu)

Validation Systems

Please, within this section, find the tables containing all the relevant information about the systems selected for validation, as well as details about the preparation of the simulation boxes.

Supporting Table S1. Description of the simulation boxes generated for each of the systems evaluated. From left to right, PDB ID, target protein family name, ligand ID, 2D representation of the ligand's chemical structure, experimental binding affinity (K_i), number of ligand copies and dimension of the system simulation box in cartesian coordinates (for X, Y and Z axes).

	PDB ID	Target Protein	Ligand ID	Ligand 2D Structure	K_i (μM)	# Ligand Copies	Dimension [XYZ] (Å)
SET I	4HW3 [52]	Myeloid cell leukemia 1 (MCL-1)	C1G (Class I)	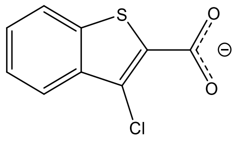	131	34	72.587<br>70.840<br>71.245
			19G (Class II)	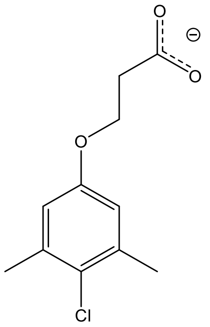	60	17	72.147<br>70.612<br>70.867
SET II	1F5K [54]	Urokinase-type plasminogen activator (uPA)	BEN	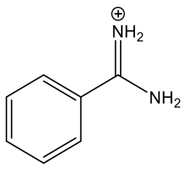	100	10	89.747<br>93.544<br>102.735
	4FU8 [54]		2UP	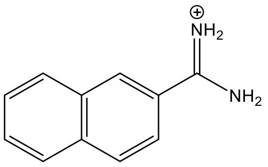	6	16	89.581<br>93.600<br>102.820
	4FUD [54]		6UP	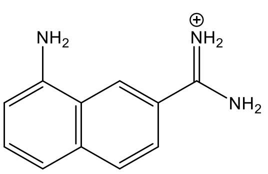	0.5	17	89.895<br>93.981<br>103.122

SET III	1I06 [61]	Major urinary protein I (MUP-I)	TZL	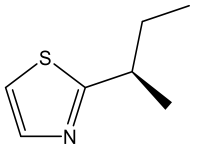	No data	17	89.635<br>88.027<br>86.870
	5YE8 [55]	Platelet-activating factor acetylhydrolase (Lp-PLA2)	8U3	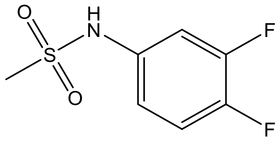	1000	20	96.980<br>96.646<br>93.221
	1W7H [56]	Mitogen-activated protein kinase 14 (MAPK14)	3IP	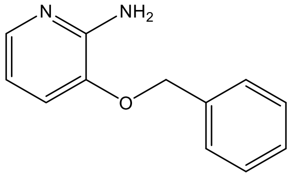	100-1000	16	104.959<br>108.906<br>105.270
	4CR5 [57]	Activated Coagulation factor (FXIa)	0UT	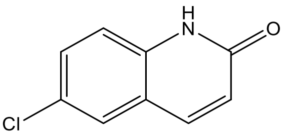	140	33	75.773<br>77.349<br>84.124
	5T4U [58]	Bromodomain and PHD Finger-containing 1 (BRPF1)	12Q	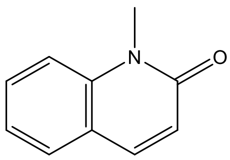	4.8	25	66.180<br>78.053<br>61.644
	3E63 [59]	Tyrosine-protein Janus Kinase (JAK2)	5B2	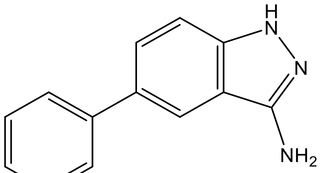	1.6	15	99.555<br>99.993<br>91.619
	3NUN [60]	Alpha Phosphoinositide - dependent Kinase - 1 (PDK1)	JMZ	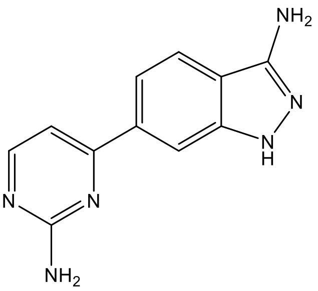	0.37	22	63.337<br>69.408<br>83.443

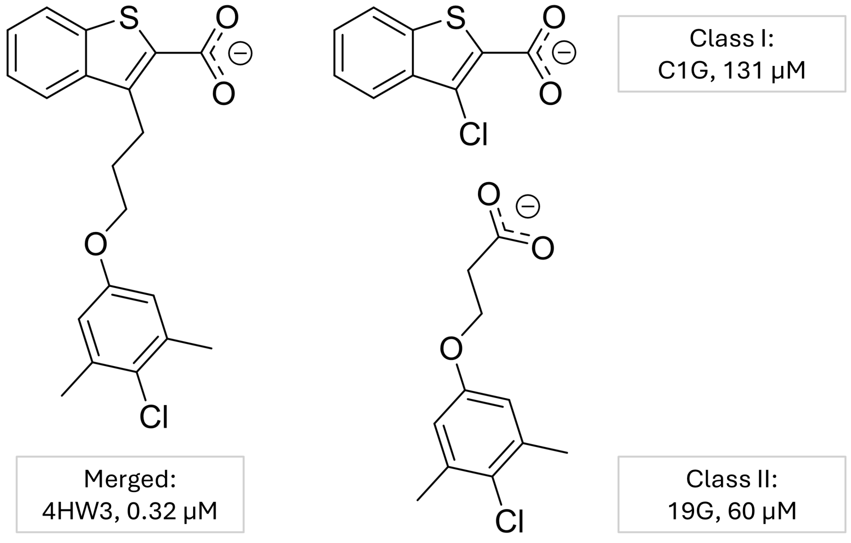

Supporting Figure S1. 2D representation of myeloid cell leukemia 1 (MCL-1) inhibitors: small fragments 3-chlorobenzo[b]thiophene-2-carboxylate (class I) and 3-(4-chloro-3,5-dimethylphenoxy)propanoate (class II) merged by linking strategy to form the larger ligand 3-[3-(4-chloro-3,5-dimethylphenoxy)propyl]-1-benzothiophene-2-carboxylic acid, with PDB ID 4WH3.

Descriptors

Please, within this section, find the tables containing the data obtained from the individual trajectory descriptors analysis when applying the methodology presented in this work. Results for each system are detailed below.

Table S2. Binding sites identified, at the 4 independent fdGaMD simulation replicates of 400 ns each, for ligand C1G of the **Class I** MCL-1 system with PDB **4HW3**. From left to right, descriptors correspond to: binding site (BS) identification label; number of "reactive" trajectories for each specific binding site (being 4, the number of replicates, the maximum possible); best Residence Time (RT) of the binding, in ns; best and averaged values for the electrostatic and vdW energy contributions (E_{inter}) and Molecular Mechanics Generalized-Born Surface Area (MMGBSA) calculated, for the last 20 ns of simulation (last) and during the residence time (RT) for each binding site, both in kcal/mol; the percentage of ligand exposed solvent accessible surface area (SASA) during the RT and at the last snapshot of the simulation; highest number of hydrogen bonds involved in the ligand-receptor interaction, for that specific binding site; and count of best final descriptor values per site (RANK). Averaged values correspond to the summatory obtained along the independent replicates for a certain descriptor and a binding site, divided by the total number of independent replicates performed. Binding site corresponding to the crystallographic information available marked with an asterisk (*). Most favorable binding site, exhibiting the best ranking position, marked in bold. Protein residues of each binding site identified for the 4HW3 system are defined in Supporting Table S23.

BS	# React.	Best RT	BEST				AVERAGED				% SASA^{RT}	% SASA^{Last}	Best # HB	RANK †
			Avg. E_{inter}^{RT}	Avg. E_{inter}^{Last}	Avg. MMGBSA^{RT}	Avg. MMGBSA^{Last}	Avg. E_{inter}^{RT}	Avg. E_{inter}^{Last}	Avg. MMGBSA^{RT}	Avg. MMGBSA^{Last}				
**BS1***	3	400	−67.52	−72.17	−23.91	−24.52	−48.24	−51.99	−16.88	−17.16	15.4	8.4	2	**8/13**
BS2	2	290	−72.27	−75.56	−17.94	−18.75	−34.64	−37.08	−8.83	−9.18	32.9	28.5	1	0/13
BS3	2	170	−83.92	−74.65	−24.85	−23.21	−41.42	−36.30	−12.30	−11.42	8.8	5.4	1	3/13
BS4	1	60	−59.03	−66.00	−14.60	−14.65	−14.76	−16.50	−3.65	−3.66	53.4	48.9	0	0/13
BS5	1	140	−29.19	−36.58	−11.20	−11.07	−7.30	−9.15	−2.80	−2.77	65.0	61.6	0	0/13
BS6	1	80	−23.47	−42.13	−14.35	−17.90	−5.87	−10.53	−3.59	−4.48	38.0	43.3	0	0/13
BS7	1	50	−87.81	−97.62	−16.82	−18.82	−21.95	−24.41	−4.21	−4.71	41.9	80.8	0	2/13

† Ranking corresponds to the number of best descriptor values, being the maximum the total number of descriptors (13/13).

Table S3. Binding sites identified, at the 4 fdGaMD simulation independent replicates of 400 ns each, for ligand 19G of the **Class II** MCL-1 system with PDB **4HW3**. Descriptors and units are defined in Supporting Table S2 caption. Binding site corresponding to the crystallographic information available marked with an asterisk (*). Most favorable binding site, exhibiting the best ranking position, marked in bold. Protein residues of each binding site identified for the 4HW3 system are defined in Supporting Table S23.

BS	# React.	Best RT	BEST				AVERAGED				% SASA^{RT}	% SASA^{Last}	Best # HB	RANK †
			Avg. E_{inter}^{RT}	Avg. E_{inter}^{Last}	Avg. MMGBSA^{RT}	Avg. MMGBSA^{Last}	Avg. E_{inter}^{RT}	Avg. E_{inter}^{Last}	Avg. MMGBSA^{RT}	Avg. MMGBSA^{Last}				
**BS1***	4	400	−56.58	−50.57	−24.47	−26.42	−52.40	−49.04	−22.69	−23.03	24.2	10.8	0	**11/13**
BS8	1	70	−49.39	−71.72	−12.6	−16.37	−12.35	−17.93	−3.15	−4.09	41.3	76.6	0	1/13
BS6	2	150	−59.88	−62.5	−22.25	−21.94	−23.99	−25.70	−10.25	−10.59	35.2	40.2	0	1/13
BS4	2	130	−77.69	−80.29	−24.42	−25.72	−37.82	−32.30	−12.13	−11.77	31.2	16.4	0	2/13
BS9	1	170	−77.39	−88.8	−17.11	−19.77	−19.35	−22.2	−4.28	−4.94	37.0	51.5	0	2/13

† Ranking corresponds to the number of best descriptor values, being the maximum the total number of descriptors (13/13).

Table S4. Binding sites identified, at the 4 fdGaMD simulation independent replicates of 400 ns each, for the urokinase system with PDB ID **1F5K**. Descriptors and units are defined in Supporting Table S2 caption. Binding site corresponding to the crystallographic information available marked with an asterisk (*). Most favorable binding site, exhibiting the best ranking position, marked in bold. Protein residues of each binding site identified for the uPA urokinase systems (set-II) are defined in Supporting Table S24.

BS	# React.	Best RT	BEST				AVERAGED				% SASA^{RT}	% SASA^{Last}	Best # HB	RANK †
			Avg. E_{inter}^{RT}	Avg. E_{inter}^{Last}	Avg. MMGBSA^{RT}	Avg. MMGBSA^{Last}	Avg. E_{inter}^{RT}	Avg. E_{inter}^{Last}	Avg. MMGBSA^{RT}	Avg. MMGBSA^{Last}				
**BS1***	1	60	−113.97	−112.69	−29.84	−30.00	−28.49	−28.17	−7.46	−7.50	8.0	24.6	3	**8/13**
BS2	1	80	−117.15	−117.73	−17.81	−18.23	−29.29	−29.43	−4.45	−4.56	67.4	68.8	3	7/13

† Ranking corresponds to the number of best descriptor values, being the maximum, the total number of descriptors (13).

Table S5. Binding sites identified, at the 4 **conventional fdMD** simulation independent replicates of 400 ns each, for the uPA urokinase system with PDB ID **1F5K.** Descriptors and units are defined in Supporting Table S2 caption. Binding site corresponding to the crystallographic information available marked with an asterisk (*). Most favorable binding site, exhibiting the best ranking position, marked in bold. Protein residues of each binding site identified for the uPA urokinase systems (validation set-II) are defined in Supporting Table S24.

BS	# React.	Best RT	BEST				AVERAGED				% SASA^{RT}	% SASA^{Last}	Best # HB	RANK †
			Avg. E_{inter}^{RT}	Avg. E_{inter}^{Last}	Avg. MMGBSA^{RT}	Avg. MMGBSA^{Last}	Avg. E_{inter}^{RT}	Avg. E_{inter}^{Last}	Avg. MMGBSA^{RT}	Avg. MMGBSA^{Last}				
BS2	1	180	−108.27	−110.53	−16.09	−15.19	−27.07	−27.63	−4.02	−3.80	65.7	62.5	3	-

† Ranking corresponds to the number of best descriptor values, being the maximum the total number of descriptors (13/13).

Table S6. Binding sites identified, at the 4 **conventional fdMD** simulation independent replicates of 400 ns each, for the uPA urokinase system with PDB ID **1F5K**. In this case, results correspond to the application of a less restrictive residence time (RT) **threshold of 20 ns.** Please note that binding site assignation may differ from the previous analysis of the same system. Descriptors and units are defined in Supporting Table S2 caption. Binding site corresponding to the crystallographic information available marked with an asterisk (*). Most favorable binding site, exhibiting the best ranking position, marked in bold. Protein residues of each binding site identified for the uPA urokinase systems (validation set-II) are defined in Supporting Table S24.

BS	# React.	Best RT	BEST				AVERAGED				% SASA^{RT}	% SASA^{Last}	Best # HB	RANK †
			Avg. E_{inter}^{RT}	Avg. E_{inter}^{Last}	Avg. MMGBSA^{RT}	Avg. MMGBSA^{Last}	Avg. E_{inter}^{RT}	Avg. E_{inter}^{Last}	Avg. MMGBSA^{RT}	Avg. MMGBSA^{Last}				
BS1*	1	30	−108.13	−113.41	−29.62	−30.40	−27.03	−28.35	−7.41	−7.60	9.8	13.0	4	5/13
**BS2**	3	180	−117.66	−117.62	−17.72	−17.69	−83.76	−85.78	−12.82	−12.60	65.7	62.5	3	**8/13**
BS3	1	20	−102.00	−102.01	−14.81	−14.83	−25.50	−25.51	−3.70	−3.71	49.0	42.2	0	0/13

† Ranking corresponds to the number of best descriptor values, being the maximum the total number of descriptors (13/13).

Table S7. Binding sites identified, at the 4 fdGaMD simulation independent replicates of 400 ns each, for the uPA urokinase system with PDB ID **4UF8**. Descriptors and units are defined in Supporting Table S2 caption. Binding site corresponding to the crystallographic information available marked with an asterisk (*). Most favorable binding site, exhibiting the best ranking position, marked in bold. Protein residues of each binding site identified for the uPA urokinase systems (validation set-II) are defined in Supporting Table S24.

BS	# React.	Best RT	BEST				AVERAGED				% SASA^{RT}	% SASA^{Last}	Best # HB	RANK †
			Avg. E_{inter}^{RT}	Avg. E_{inter}^{Last}	Avg. MMGBSA^{RT}	Avg. MMGBSA^{Last}	Avg. E_{inter}^{RT}	Avg. E_{inter}^{Last}	Avg. MMGBSA^{RT}	Avg. MMGBSA^{Last}				
**BS1***	1	90	−118.03	−115.88	−36.00	−36.01	−29.51	−28.97	−9.00	−9.00	14.0	15.6	3	**12/13**
BS2	1	100	−109.44	−110.98	−18.56	−19.20	−27.36	−27.75	−4.64	−4.80	61.7	60.3	3	2/13
BS4	1	50	−63.73	−72.45	−12.36	−13.67	−15.93	−18.11	−3.09	−3.42	62.4	64.9	0	0/13
BS5	1	350	−43.40	−46.03	−18.26	−20.19	−10.85	−11.51	−4.57	−5.05	26.7	28.3	0	2/13

† Ranking corresponds to the number of best descriptor values, being the maximum the total number of descriptors (13/13).

Table S8. Binding sites identified, at the 4 **conventional fdMD** simulation independent replicates of 400 ns each, for the uPA urokinase system with PDB ID **4UF8**. Descriptors and units are defined in Supporting Table S2 caption. Binding site corresponding to the crystallographic information available marked with an asterisk (*). Most favorable binding site, exhibiting the best ranking position, marked in bold. Protein residues of each binding site identified for the uPA urokinase systems (validation set-II) are defined in Supporting Table S24.

BS	# React.	Best RT	BEST				AVERAGED				% SASA^{RT}	% SASA^{Last}	Best # HB	RANK †
			Avg. E_{inter}^{RT}	Avg. E_{inter}^{Last}	Avg. MMGBSA^{RT}	Avg. MMGBSA^{Last}	Avg. E_{inter}^{RT}	Avg. E_{inter}^{Last}	Avg. MMGBSA^{RT}	Avg. MMGBSA^{Last}				
BS3	1	60	−33.56	−65.04	−12.27	−14.15	−8.39	−16.26	−3.07	−3.74	51.5	46.4	0	-

† Ranking corresponds to the number of best descriptor values, being the maximum the total number of descriptors (13/13).

Table S9. Binding sites identified, at the 4 fdGaMD simulation independent replicates of 400 ns each, for the uPA urokinase system with PDB ID **4UFD**. Descriptors and units are defined in Supporting Table S2 caption. Binding site corresponding to the crystallographic information available marked with an asterisk (*). Most favorable binding site, exhibiting the best ranking position, marked in bold. Protein residues of each binding site identified for the uPA urokinase systems (validation set-II) are defined in Supporting Table S24.

BS	# React.	Best RT	BEST				AVERAGED				% SASA^{RT}	% SASA^{Last}	Best # HB	RANK†
			Avg. E_{inter}^{RT}	Avg. E_{inter}^{Last}	Avg. MMGBSA^{RT}	Avg. MMGBSA^{Last}	Avg. E_{inter}^{RT}	Avg. E_{inter}^{Last}	Avg. MMGBSA^{RT}	Avg. MMGBSA^{Last}				
BS2	1	200	−97.19	−97.46	−19.03	−19.51	−24.30	−24.37	−4.76	−4.88	45.0	42.3	2	0/13
**BS1***	1	210	−121.48	−122.29	−36.05	−36.33	−30.37	−30.57	−9.01	−9.08	16.6	14.1	4	**13/13**
BS6	1	70	−28.07	−28.70	−12.52	−13.20	−7.018	−7.18	−3.13	−3.30	49.5	52.1	0	0/13

† Ranking corresponds to the number of best descriptor values, being the maximum the total number of descriptors (13/13).

Table S10. Binding sites identified, at the 4 **conventional fdMD** simulation independent replicates of 400 ns each, for the uPA urokinase system with PDB ID **4UFD**. Descriptors and units are defined in Supporting Table S2 caption. Binding site corresponding to the crystallographic information available marked with an asterisk (*). Most favorable binding site, exhibiting the best ranking position, marked in bold. Protein residues of each binding site identified for the uPA urokinase systems (validation set-II) are defined in Supporting Table S24.

BS	# React.	Best RT	BEST				AVERAGED				% SASA^{RT}	% SASA^{Last}	Best # HB	RANK†
			Avg. E_{inter}^{RT}	Avg. E_{inter}^{Last}	Avg. MMGBSA^{RT}	Avg. MMGBSA^{Last}	Avg. E_{inter}^{RT}	Avg. E_{inter}^{Last}	Avg. MMGBSA^{RT}	Avg. MMGBSA^{Last}				
BS	1	70	−50.8	−78.26	−10.24	−11.63	−12.70	−19.57	−2.56	−2.91	63.3	53.4	0	0/13
**BS2**	2	160	−109.31	−105.31	−18.17	−18.47	−49.24	−48.87	−9.04	−8.82	49.0	41.5	3	**9/13**
BS1*	1	90	−42.51	−45.6	−20.05	−20.78	−10.63	−11.40	−5.01	−5.20	31.1	30.2	0	4/13

† Ranking corresponds to the number of best descriptor values, being the maximum the total number of descriptors (13/13).

Table S11. Binding sites identified, at the 4 fdGaMD simulation independent replicates of 400 ns each, for the MUP-I system with PDB ID **1I06**. Descriptors and units are defined in Supporting Table S2 caption. Binding site corresponding to the crystallographic information available marked with an asterisk (*). Most favorable binding site, exhibiting the best ranking position, marked in bold. Protein residues of each binding site identified for the system are defined in Supporting Table S25.

BS	# React.	Best RT	BEST				AVERAGED				% SASA^{RT}	% SASA^{Last}	Best # HB	RANK†
			Avg. E_{inter}^{RT}	Avg. E_{inter}^{Last}	Avg. MMGBSA^{RT}	Avg. MMGBSA^{Last}	Avg. E_{inter}^{RT}	Avg. E_{inter}^{Last}	Avg. MMGBSA^{RT}	Avg. MMGBSA^{Last}				
BS1	1	220	−14.93	−14.69	−10.14	−9.94	−3.73	−3.67	−2.54	−2.49	54.2	65.5	0	-

† Ranking corresponds to the number of best descriptor values, being the maximum the total number of descriptors (13/13).

Table S12. Binding sites identified, at the 4 fdGaMD simulation independent replicates of 400 ns each, for the MUP-I system with PDB ID **1I06**. In this case, results correspond to the application of a less restrictive residence time (RT) **threshold of 20 ns**. Please note that binding site assignation may differ from the previous analysis of the same system. Descriptors and units are defined in Supporting Table S2 caption. Binding site corresponding to the crystallographic information available marked with an asterisk (*). Most favorable binding site, exhibiting the best ranking position, marked in bold. Protein residues of each binding site identified for the system are defined in Supporting Table S25.

BS	# React.	Best RT	BEST				AVERAGED				% SASA^{RT}	% SASA^{Last}	Best # HB	RANK †
			Avg. E_{inter}^{RT}	Avg. E_{inter}^{Last}	Avg. MMGBSA^{RT}	Avg. MMGBSA^{Last}	Avg. E_{inter}^{RT}	Avg. E_{inter}^{Last}	Avg. MMGBSA^{RT}	Avg. MMGBSA^{Last}				
**BS2***	1	30	−24.44	−24.68	−19.8	−20.06	−6.11	−6.17	−4.95	−5.02	0.8	0.0	0	**9/13**
BS1	2	220	−14.93	−15.25	−10.14	−9.94	−7.21	−7.49	−4.71	−4.88	53.9	36.0	0	3/13
BS3	1	40	−16.92	−20.45	−10.28	−11.72	−4.23	−5.11	−2.57	−2.93	40.9	48.0	0	0/13

† Ranking corresponds to the number of best descriptor values, being the maximum the total number of descriptors (13/13).

Table S13. Final descriptor values obtained by binding sites, for Lp-PLA2 system with PDB ID **5YE8**, at the 4 fdGaMD simulation independent replicates of 400 ns each. Descriptors and units are defined in Supporting Table S2 caption. Binding site corresponding to the crystallographic information available marked with an asterisk (*). Most favorable binding site, exhibiting the best ranking position, marked in bold. Protein residues of each binding site are defined in Supporting Table S26.

BS	# React.	Best RT	BEST				AVERAGED				% SASA^{RT}	% SASA^{Last}	Best # HB	RANK †
			Avg. E_{inter}^{RT}	Avg. E_{inter}^{Last}	Avg. MMGBSA^{RT}	Avg. MMGBSA^{Last}	Avg. E_{inter}^{RT}	Avg. E_{inter}^{Last}	Avg. MMGBSA^{RT}	Avg. MMGBSA^{Last}				
BS1	1	80	−23.59	−27.19	−10.31	−10.03	−5.90	−6.80	−2.58	−2.51	58.8	52.6	0	0/13
**BS2***	2	400	−36.77	−39.01	−17.81	−21.33	−17.55	−17.71	−8.36	−9.14	8.2	0.0	1	**8/13**
BS3	1	110	−30.83	−32.44	−15.29	−15.82	−7.71	−8.11	−3.82	−3.96	40.6	36.9	0	0/13
BS4	1	140	−54.33	−57.80	−18.29	−19.74	−13.58	−14.45	−4.57	−4.94	26.0	55.1	0	2/13
BS5	1	50	−43.42	−47.49	−20.46	−21.52	−10.86	−11.87	−5.12	−5.38	29.3	58.9	2	3/13

† Ranking corresponds to the number of best descriptor values, being the maximum the total number of descriptors (13/13).

Table S14. Binding sites identified, at the 4 fdGaMD simulation independent replicates of 400 ns each, for MAPK14 system with PDB ID **1WH7**. Descriptors and units are defined in Supporting Table S2 caption. Binding site corresponding to the crystallographic information available marked with an asterisk (*). Most favorable binding site, exhibiting the best ranking position, marked in bold. Protein residues of each binding site are defined in Supporting Table S27.

BS	# React.	Best RT	BEST				AVERAGED				% SASA^{RT}	% SASA^{Last}	Best # HB	RANK †
			Avg. E_{inter}^{RT}	Avg. E_{inter}^{Last}	Avg. MMGBSA^{RT}	Avg. MMGBSA^{Last}	Avg. E_{inter}^{RT}	Avg. E_{inter}^{Last}	Avg. MMGBSA^{RT}	Avg. MMGBSA^{Last}				
BS1	3	260	−25.29	−25.59	−15.26	−15.88	−18.57	−18.53	−11.02	−11.21	42.6	46.3	0	0/13
**BS2***	4	310	−46.42	−46.43	−31.30	−31.36	−42.60	−42.68	−27.99	−28.03	7.4	0.0	1	**13/13**
BS3	1	130	−28.62	−30.43	−16.25	−17.76	−7.16	−7.61	−4.06	−4.44	43.4	50.5	0	0/13
BS4	1	80	−28.41	−27.87	−14.87	−14.48	−7.10	−6.97	−3.72	−3.62	48.7	54.2	0	0/13
BS5	1	300	−38.42	−43.59	−20.17	−22.35	−9.61	−10.90	−5.04	−5.59	17.7	22.3	0	0/13

† Ranking corresponds to the number of best descriptor values, being the maximum the total number of descriptors (13/13).

Table S15. Binding sites identified, at the 4 fdGaMD simulation independent replicates of 400 ns each, for FXIa system with PDB ID **4CR5**. Descriptors and units are defined in Supporting Table S2 caption. Binding site corresponding to the crystallographic information available marked with an asterisk (*). Most favorable binding site, exhibiting the best ranking position, marked in bold. Protein residues of each binding site are defined in Supporting Table S28.

BS	# React.	Best RT	BEST				AVERAGED				% SASA^{RT}	% SASA^{Last}	Best # HB	RANK †
			Avg. E_{inter}^{RT}	Avg. E_{inter}^{Last}	Avg. $MMGBSA^{RT}$	Avg. $MMGBSA^{Last}$	Avg. E_{inter}^{RT}	Avg. E_{inter}^{Last}	Avg. $MMGBSA^{RT}$	Avg. $MMGBSA^{Last}$				
**BS1**	3	290	−40.21	−40.89	−23.36	−24.03	−24.33	−27.03	−13.77	−14.28	6.9	3.6	0	**6/13**
BS2	4	310	−27.65	−27.43	−17.06	−16.72	−24.94	−25.42	−14.74	−14.15	40.0	39.4	0	3/13
BS3	3	400	−26.57	−33.02	−18.43	−19.55	−18.20	−21.83	−10.43	−11.65	20.7	24.9	0	1/13
BS4*	1	110	−38.34	−38.11	−22.78	−22.92	-9.59	−9.53	−5.70	−5.73	20.5	19.1	0	0/13
BS5	2	60	−41.76	−42.16	−24.84	−25.03	−17.47	−17.81	−10.32	−10.49	26.1	16.8	2	2/13
BS6	2	70	−18.93	−19.12	−10.76	−11.25	−12.34	−11.99	−7.55	−7.63	49.8	58.6	1	0/13
BS7	1	340	−37.38	−36.92	−24.68	−25.60	−9.35	−9.23	−6.17	−6.40	33.0	29.0	1	1/13
BS8	1	230	−24.41	−24.13	−14.41	−13.93	−6.10	−6.03	−3.60	−3.48	50.0	38.2	0	0/13

† Ranking corresponds to the number of best descriptor values, being the maximum the total number of descriptors (13/13).

Table S16. Binding sites identified, at the 4 fdGaMD simulation independent replicates of 400 ns each, for BRPF1 system with PDB ID **5T4U**. Descriptors and units are defined in Supporting Table S2 caption. Binding site corresponding to the crystallographic information available marked with an asterisk (*). Most favorable binding site, exhibiting the best ranking position, marked in bold. Protein residues of each binding site are defined in Supporting Table S29.

BS	# React.	Best RT	BEST				AVERAGED				% SASA^{RT}	% SASA^{Last}	Best # HB	RANK †
			Avg. E_{inter}^{RT}	Avg. E_{inter}^{Last}	Avg. $MMGBSA^{RT}$	Avg. $MMGBSA^{Last}$	Avg. E_{inter}^{RT}	Avg. E_{inter}^{Last}	Avg. $MMGBSA^{RT}$	Avg. $MMGBSA^{Last}$				
**BS1***	3	400	−40.95	−41.38	−20.55	−20.88	−27.17	−26.65	−14.24	−13.76	25.4	24.0	1	**11/13**
BS2	1	210	−28.98	−28.99	−20.65	−21.00	−7.25	−7.25	−5.16	−5.25	11.9	10.5	0	2/13
BS3	1	110	−21.60	−23.14	−13.69	−13.95	−5.40	−5.79	−3.42	−3.49	41.3	43.3	0	0/13

† Ranking corresponds to the number of best descriptor values, being the maximum the total number of descriptors (13/13).

Table S17. Binding sites identified, at the 4 fdGaMD simulation independent replicates of 400 ns each, for JAK2 system with PDB ID **3E63**. Descriptors and units are defined in Supporting Table S2 caption. Binding site corresponding to the crystallographic information available marked with an asterisk (*). Most favorable binding site, exhibiting the best ranking position, marked in bold. Protein residues of each binding site are defined in Supporting Table S30.

BS	# React.	Best RT	BEST				AVERAGED				% SASA^{RT}	% SASA^{Last}	Best # HB	RANK †
			Avg. E_{inter}^{RT}	Avg. E_{inter}^{Last}	Avg. MMGBSA^{RT}	Avg. MMGBSA^{Last}	Avg. E_{inter}^{RT}	Avg. E_{inter}^{Last}	Avg. MMGBSA^{RT}	Avg. MMGBSA^{Last}				
BS1	1	80	−28.78	−36.65	−12.47	−15.10	−7.2	−9.16	−3.12	−3.78	53.9	47.1	0	0/13
**BS2***	4	250	−46.13	−46.64	−26.69	−26.76	−45.17	−44.97	−25.82	−25.84	14.2	14.4	2	**9/13**
BS3	1	60	−23.02	−26.96	−11.66	−13.97	−5.76	−6.74	−2.92	−3.49	52.8	44.3	0	0/13
BS4	1	320	−29.89	−30.14	−15.93	−15.12	−7.47	−7.54	−3.98	−3.78	52.7	45.9	0	1/13
BS5	1	70	−42.29	−43.22	−24.36	−25.46	−10.57	−10.81	−6.09	−6.37	11.3	16.2	0	1/13
BS6	1	70	−55.63	−56.59	−24.01	−24.51	−13.91	−14.15	−6.00	−6.13	27.0	33.6	1	2/13
BS7	1	140	−24.05	−25.07	−12.73	−13.47	−6.01	−6.27	−3.18	−3.37	57.2	51.3	0	0/13
BS8	1	220	−25.02	−25.39	−13.20	−13.81	−6.26	−6.35	−3.30	−3.45	50.2	56.5	0	0/13
BS9	1	80	−28.78	−36.65	−12.47	−15.10	−7.20	−9.16	−3.12	−3.78	53.9	47.1	0	0/13

† Ranking corresponds to the number of best descriptor values, being the maximum the total number of descriptors (13/13).

Table S18. Binding sites identified, at the 4 fdGaMD simulation independent replicates of 400 ns each, for the PDK-1 system with PDB **3NUN**. Descriptors and units are defined in Supporting Table S2 caption. Binding site corresponding to the crystallographic information available marked with an asterisk (*). Most favorable binding site, exhibiting the best ranking position, marked in bold. Protein residues of each binding site are defined in Supporting Table S31.

BS	# React.	Best RT	BEST				AVERAGED				% SASA^{RT}	% SASA^{Last}	Best # HB	RANK †
			Avg. E_{inter}^{RT}	Avg. E_{inter}^{Last}	Avg. MMGBSA^{RT}	Avg. MMGBSA^{Last}	Avg. E_{inter}^{RT}	Avg. E_{inter}^{Last}	Avg. MMGBSA^{RT}	Avg. MMGBSA^{Last}				
BS1	1	160	−43.92	−48.11	−25.58	−26.27	−10.98	−12.03	−6.40	−6.57	20.7	20.3	1	0/13
BS2	1	200	−42.77	−43.43	−22.32	−22.71	−10.69	−10.86	−5.58	−5.68	22.0	41.2	0	0/13
BS3	1	280	−32.76	−32.90	−14.51	−13.64	−8.19	−8.23	−3.63	−3.41	53.7	58.4	1	0/13
BS4	3	170	−44.47	−46.64	−22.64	−22.64	−28.73	−29.47	−13.88	−13.56	22.7	18.7	1	1/13
BS5	2	60	−35.56	−37.47	−16.95	−17.97	−17.21	−18.13	−7.44	−7.90	34.5	34.6	0	0/13
BS6	1	80	−33.16	−37.39	−14.92	−15.91	−8.29	−9.35	−3.73	−3.98	45.7	38.4	0	0/13
BS7	3	220	−64.08	−62.56	−28.27	−28.20	−39.52	−40.15	−16.94	−17.01	11.8	15.8	1	4/13
BS8	2	340	−37.21	−37.21	−17.46	−17.34	−17.67	−17.92	−8.70	−8.45	50.2	34.5	1	1/13
**BS9***	3	260	−67.01	−66.64	−32.45	−33.02	−40.55	−39.36	−18.79	−18.58	12.6	9.1	3	**11/13**
BS10	1	170	−45.38	−45.52	−17.02	−17.27	−11.35	−11.38	−4.26	−4.32	53.5	44.5	1	0/13
BS11	1	60	−30.20	−28.91	−12.7	−12.63	−7.55	−7.23	−3.18	−3.16	54.3	64.8	0	0/13
BS12	2	70	−49.76	−49.76	−19.6	−19.63	−24.04	−24.32	−9.62	−9.81	36.7	37.0	0	0/13
BS13	3	100	−49.88	−49.29	−18.24	−18.62	−33.17	−33.46	−12.02	−12.06	48.6	54.9	1	1/13
BS14	2	290	−32.72	−32.72	−13.42	−13.32	−15.82	−15.61	−5.64	−6.50	63.3	62.1	1	0/13
BS15	1	60	−38.02	−37.81	−11.77	−12.41	−9.51	−9.45	−2.94	−3.10	38.7	68.9	0	0/13
BS16	1	140	−28.18	−30.84	−12.88	−14.13	−7.05	−7.71	−3.22	−3.53	61.1	43.2	0	0/13
BS17	2	180	−50.45	−50.48	−24.75	−23.32	−22.73	−22.65	−9.78	−9.44	16.7	30.6	1	0/13
BS18	2	80	−46.32	−46.32	−16.73	−16.73	−21.57	−21.34	−7.93	−7.86	35.9	35.5	0	0/13
BS19	1	260	−41.38	−41.34	−15.85	−15.54	−10.35	−10.34	−3.96	−3.89	47.8	43.7	0	0/13

† Ranking corresponds to the number of best descriptor values, being the maximum the total number of descriptors (13/13).

Table S19. Binding sites identified, at the **12** independent fdGaMD simulation replicates of **400 ns** for urokinase system with PDB **4CR5**. Descriptors and units are defined in Supporting Table S2 caption. Binding site corresponding to the crystallographic information available marked with an asterisk (*). Most favorable binding site, exhibiting the best ranking position, marked in bold. Protein residues of each binding site are defined in Supporting Table S28.

BS	# React.	Best RT	BEST				AVERAGED				% SASA^{RT}	% SASA^{Last}	Best # HB	RANK †
			Avg. E_{inter}^{RT}	Avg. E_{inter}^{Last}	Avg. MMGBSA^{RT}	Avg. MMGBSA^{Last}	Avg. E_{inter}^{RT}	Avg. E_{inter}^{Last}	Avg. MMGBSA^{RT}	Avg. MMGBSA^{Last}				
**BS4***	4	380	−39.98	−47.69	−23.80	−24.20	−11.32	−12.30	−6.73	−7.21	9.7	0.0	1	**7/13**
BS9	2	100	−31.50	−30.17	−18.65	−18.25	−4.69	−4.54	−3.08	−3.01	17.5	18.8	0	0/13
BS10	2	140	−18.98	−19.90	−11.45	−12.68	−3.07	−3.08	−1.89	−2.00	42.4	41.6	0	0/13
BS2	3	120	−27.54	−28.99	−14.00	−14.42	−6.12	−6.13	−3.31	−3.28	45.7	40.8	1	0/13
BS8	1	70	−31.80	−37.36	−16.06	−17.87	−2.65	−3.11	−1.34	−1.49	35.4	38.8	0	0/13
BS5	5	220	−29.78	−33.84	−18.68	−21.95	−11.59	−12.03	−7.15	−7.42	28.0	9.2	0	4/13
BS7	4	300	−33.20	−32.69	−23.70	−23.96	−10.41	−10.54	−6.94	−7.00	0.7	0.0	1	1/13
BS3	3	130	−27.53	−29.94	−18.55	−19.28	−6.18	−6.49	−4.26	−4.34	28.3	23.1	0	0/13
BS1	2	130	−28.52	−28.66	−17.13	−16.32	−4.36	−4.62	−2.48	−2.59	35.7	27.7	0	0/13
BS11	1	60	−21.54	−22.37	−13.23	−14.34	−1.80	−1.86	−1.10	−1.20	64.4	54.4	0	0/13
BS12	1	70	−34.00	−33.91	−16.45	−16.37	−2.83	−2.83	−1.37	−1.36	51.2	52.2	2	1/13

† Ranking corresponds to the number of best descriptor values, being the maximum the total number of descriptors (13/13).

Table S20. Detailed descriptor analysis for all the reactive trajectories only of the experimental binding site (BS4) from the **12** independent fdGaMD simulation replicates of **400 ns** for system **4CR5**. Descriptors and units are defined in Supporting Table S2 caption. Most favorable **binding modes**, exhibiting the best ranking position, marked in bold. See Figure 5 (main manuscript) for pose details.

# DYN	Binding mode	Avg. E_{inter}^{RT}	Avg. E_{inter}^{Last}	Avg. MMGBSA^{RT}	Avg. MMGBSA^{Last}	Residence Time (RT)	% SASA^{RT}	% SASA^{Last}	# Hydrogen Bonds	RANK †
1	**Native**	−39.98	−47.69	−21.85	−23.51	380	20.4	0.0	1	**5/8**
5	Flipped	−37.17	−39.95	−21.58	−21.8	340	9.7	5.3	0	1/8
8	Backwards	−35.77	−35.95	−23.8	−24.2	130	19.9	26.2	1	3/8
10	Flipped	−22.92	−24.02	−13.55	−17.05	50	58.0	32.3	0	0/8

† Ranking corresponds to the number of best descriptor values, being the maximum the total number of descriptors (8/8).

Table S21. Binding sites identified, at the **48** independent fdGaMD simulation replicates of **100 ns** each, for the urokinase system with PDB **4CR5**. Descriptors and units are defined in Table S2 caption. Binding site corresponding to the crystallographic information available marked with an asterisk (*). Most favorable binding site, exhibiting the best ranking position, marked in bold. Protein residues of each binding site are defined in supporting table S33 and may differ from previous BS assignations.

BS	# React.	Best RT	BEST				AVERAGED				% SASA^{RT}	% SASA^{Last}	Best # HB	RANK †
			Avg. E_{inter}^{RT}	Avg. E_{inter}^{Last}	Avg. MMGBSA^{RT}	Avg. MMGBSA^{Last}	Avg. E_{inter}^{RT}	Avg. E_{inter}^{Last}	Avg. MMGBSA^{RT}	Avg. MMGBSA^{Last}				
BS13	30	100	−31.35	−31.41	−16.97	−17.59	−16.60	−17.04	−8.90	−9.11	23.2	17.3	1	5/13
BS14	15	70	−36.22	−36.22	−20.11	−20.30	−8.95	−9.18	−5.66	−5.78	10.0	6.2	1	2/13
**BS4***	17	90	−38.92	−41.08	−23.32	−23.84	−10.81	−11.57	−7.26	−7.34	8.4	6.8	1	**6/13**
BS15	2	30	−21.32	−25.13	−13.22	−13.84	−0.88	−0.96	−0.53	−0.56	46.3	46.6	0	0/13
BS16	21	100	−31.68	−31.63	−21.71	−21.57	−8.59	−8.78	−5.22	−5.30	13.6	20.3	0	1/13
BS5	27	100	−30.83	−33.62	−19.92	−21.32	−15.55	−15.64	−9.27	−9.22	26.8	9.1	1	4/13
BS8	5	70	−24.54	−25.27	−17.24	−17.19	−2.19	−2.18	−1.45	−1.46	43.9	36.5	0	0/13
BS1	9	90	−30.50	−33.00	−17.55	−18.34	−5.22	−5.41	−2.95	−3.02	21.9	10.4	0	0/13
BS17	6	90	−25.31	−28.04	−13.69	−14.41	−2.21	−2.66	−1.47	−1.58	42.7	42.4	0	0/13
BS18	5	100	−26.99	−28.32	−16.29	−16.29	−2.39	−2.50	−1.36	−1.40	38.5	35.0	1	2/13
BS19	1	20	−23.88	−27.53	−13.90	−13.90	−0.50	−0.57	−0.29	−0.29	55.9	74.7	0	0/13
BS7	2	40	−28.41	−29.00	−19.45	−19.29	−1.01	−1.07	−0.61	−0.64	19.4	20.6	0	0/13
BS20	1	50	−23.38	−23.25	−13.44	−13.08	−0.49	−0.48	−0.28	−0.27	46.6	46.7	0	0/13
BS21	3	30	−36.00	−36.00	−14.99	−16.15	−2.04	−2.06	−0.88	−0.91	23.9	31.9	1	1/13
BS22	1	30	−26.31	−26.41	−14.84	−14.84	−0.55	−0.55	−0.31	−0.31	42.9	39.1	0	0/13
BS3	1	50	−19.90	−20.00	−14.71	−14.41	−0.41	−0.42	−0.31	−0.30	34.5	25.2	0	0/13
BS23	2	50	−24.45	−24.45	−12.34	−12.34	−0.87	−0.82	−0.48	−0.48	47.0	46.9	0	0/13

† Ranking corresponds to the number of best descriptor values, being the maximum the total number of descriptors (13/13).

Table S22. Example of the **trajectory analysis** performed for any studied system, being system with PDB ID **1W7H** the one presented in this case. These results, obtained after applying the fdGaMD approach presented, are computed from the 4 independent replicates of 400 ns performed. Hence, for every "reactive" ligand trajectory (ligand identifier) for each of the independent replicates performed (# DYN), exhibiting interactions at a given binding site (BS) during a certain residence time (RT; in ns), the averaged interaction energy obtained during the RT (Avg. E_{inter}^{RT}; in kcal/mol) and the last 20 ns of simulation (Avg. E_{inter}^{LAST}; in kcal/mol), the averaged Molecular Mechanics Generalized-Born Surface Area free binding energy estimation during the RT (Avg. $MMGBSA^{RT}$; in kcal/mol) and the last 20 ns of simulations (Avg. $MMGBSA^{LAST}$; in kcal/mol), the percentage of ligand exposed solvent accessible surface area during the RT (% $SASA^{RT}$) and at the last snapshot of the simulation (% $SASA^{Last}$), as well as the determination of the number of hydrogen bonds formed during the last 20 ns of simulation, are all computed as equally weighted analysis descriptors. These results will be processed to a final binding-site based ranking, in the search of druggable hotspots for the studied fragment and target protein. Binding site corresponding to the crystallographic information available marked with an asterisk (*). Please, notice that ligand numbering is designated as following the number of protein residues. Protein residues of each binding site are defined in Supporting Table S27. Best values for each descriptor are highlighted in bold.

# DYN	Ligand identifier	Binding Site	Residence Time (RT)	Avg. E_{inter}^{RT}	Avg. E_{inter}^{Last}	Avg. $MMGBSA^{RT}$	Avg. $MMGBSA^{Last}$	% $SASA^{RT}$	% $SASA^{Last}$	# Hydrogen Bonds
1	lig_356	BS1	**260**	−24.89	−23.19	−14.02	−13.95	48.6	50.0	0
	lig_358	BS2*	240	**−41.15**	**−42.00**	**−26.49**	**−27.32**	**13.1**	**50.0**	0
2	lig_358	BS2*	**310**	**−43.56**	**−43.39**	**−28.69**	**−28.59**	**5.7**	**7.1**	**1**
	lig_360	BS3	130	−28.62	−30.43	−16.25	−17.76	43.4	50.5	0
	lig_364	BS4	80	−28.41	−27.87	−14.87	−14.48	48.7	54.2	0
	lig_368	BS1	130	−24.1	−25.59	−14.79	−15.88	42.6	56.0	0
3	lig_358	BS2*	120	**−39.25**	−38.9	**−25.49**	**−24.86**	21.9	**15.4**	0
	lig_359	BS5	**300**	−38.42	**−43.59**	−20.17	−22.35	**17.7**	22.3	0
4	lig_360	BS1	150	−25.29	−25.35	−15.26	−15.01	43.9	46.3	0
	lig_362	BS2*	**170**	**−46.42**	**−46.43**	**−31.30**	**−31.36**	**7.4**	**0.0**	**1**

Binding Sites Definition (List of Protein Residues)

This section provides the tables corresponding to all binding sites identified for the validation systems studied.

Table S23. List of protein residues defining each binding site identified across the four independent 400 ns fdGaMD simulation replicates for ligand C1G (**Class I**) and ligand 19G (**Class II**) in the system with PDB ID **4HW3**. In this system, the first residue of the simulated target (UniProtKB Q07820) corresponds to D172 in the canonical sequence. Binding site corresponding to the crystallographic information available marked with an asterisk (*).

BS	Protein Residues
BS1*	53 56 57 60 63 78 79 80 82 83 85 86 91 92 95 96 99 100 126
BS2	12 15 16 19 20 104 108 113 116 117 120
BS3	3 4 6 7 30 31 127 128 131 132 135 136 139 140 141 142
BS4	58 61 62 66 67 68 69 72 75 102 103 104 106 107 110 112 115 119
BS5	33 36 37 40 44 150
BS6	69 72 73 75 76 107 115 118 119 122
BS7	73 74 76 77 79 80 121 122 123 125 126
BS8	60 63 64 74 77 78 81 82
BS9	45 49 52 53 86 87 89 91 92 94 95 147 148

Table S24. List of protein residues defining each binding site identified across the four independent 400 ns fdMD and fdGaMD simulation replicates for the uPA urokinase set-II systems with PDB ID **1F5K**, **4FU8** and **4FUD**. In this system, the first residue of the simulated target (UniProtKB P00749) corresponds to I1 in the canonical sequence. Binding site corresponding to the crystallographic information available marked with an asterisk (*).

BS	Protein Residues
BS1*	46 92 137 192 193 194 195 197 198 216 217 218 219 220 221 222 223 224 227 228 229 230 231
BS2	10 11 12 13 14 118 133 134 135 136 138 156 158 203 204 205 210
BS3	88 89 90 91 92 93 95 173 174 176 177 178 181 218 220 230
BS4	4 5 156 157 158 159 186 187 188 191
BS5	123 124 127 128 129 130 133 203 204 205 206 207 211 212 213 235
BS6	28 29 66 67 142 147 148 149 150 195
BS7	6 143 144 147 148 149 150 151 152 153

Table S25. List of protein residues defining each binding site identified across the four independent 400 ns fdGaMD simulation replicates for system with PDB ID **1I06**. In this system, the first residue of the simulated target (UniProtKB P02762) corresponds to E19 in the canonical sequence. Binding site corresponding to the crystallographic information available marked with an asterisk (*).

BS	Protein Residues
BS1	23 128 131 132 135 145 146 148 149 150
BS2*	24 38 40 42 45 54 56 69 82 84 90 91 101 103 104 105 116 117 118 120
BS3	39 41 57 59 62 63 64 154 155 156

Table S26. List of protein residues defining each binding site identified across the four independent 400 ns fdGaMD simulation replicates for system with PDB **5YE8**. In this system, the first residue of the simulated target (UniProtKB Q13093) corresponds to K55 in the canonical sequence. Binding site corresponding to the crystallographic information available marked with an asterisk (*).

BS	Protein Residues
BS1	161 162 165 169 246 247 248 249 276
BS2*	53 56 57 97 98 99 100 101 102 105 106 131 218 219 220 297 298 299 301 303 317
BS3	185 188 189 190 191 192 193 201 202 204 205
BS4	303 304 305 307 308 309 312 313 317 318 319 322 323 324
BS5	47 50 51 54 59 60 61 62 63 64 65 66 68

Table S27. List of protein residues defining each binding site identified across the four independent 400 ns fdGaMD simulation replicates for system with PDB **1W7H**. In this system, the first residue of the simulated target (UniProtKB Q16539) corresponds to E4 in the canonical sequence. Binding site corresponding to the crystallographic information available marked with an asterisk (*).

BS	Protein Residues
BS1	46 106 107 108 113 117 119 120 123 127 155 156 157 158 159 160
BS2*	27 28 32 35 48 49 50 52 68 72 81 83 86 101 102 103 104 105 106 107 108 154 162 164 165 168
BS3	10 11 12 13 15 26 27 28 29 33 34 35 36
BS4	230 231 233 234 259 260 261 262
BS5	67 71 143 144 145 146 147 166 167 168 170 171 176 320 322 325

Table S28. List of protein residues defining each binding site identified across the different independent 400 ns and 100 ns fdGaMD simulation replicates for system with PDB ID **4CR5**. In this system, the first residue of the simulated target (UniProtKB P03951) corresponds to I16 in the canonical sequence. Binding site corresponding to the crystallographic information available is marked with an asterisk (*).

BS	Protein Residues
BS1	44 87 88 89 90 91 93 160 161 162 165 166 167 171 207 208 209 210 211 217 218 219 220 221 222
BS2	19 25 26 27 28 64 65 67 132 133 134 135 139 140 141 142 143 185 186 208
BS3	151 153 157 160 161 162 163 174 175 176 177 214 215 216 217 218 219
BS4*	2 44 136 137 182 183 184 185 186 187 188 189 206 207 208 209 210 211 212 213 214 215 218 219 220 221
BS5	115 116 117 118 119 120 121 122 123 124 125 127 152 153 154 155 156 169 172 173 194 196 201 203 223 225 226
BS6	34 35 36 38 112 113 114 117 231 232 235
BS7	16 33 35 36 37 38 39 57 58 59 60 74 99 102 103 104 105 106 107 108 109 110 111
BS8	2 3 134 135 136 137 138 139 184 185 209 210 211 212 213 214
BS9	18 20 28 45 46 49 51 52 53 54 55 56 57 76 77 78 79 97
BS10	34 35 36 38 96 114 115 116 228 229 231 232 235 236 237 238
BS11	57 58 59 60 61 72 73 74 104 106 109
BS12	1 2 3 4 5 6 133 134 135 139 140 142 146 147
BS13	17 19 21 25 26 27 56 58 64 65 67 68 69 132 140 141 142 143
BS14	10 13 14 33 34 35 36 59 60 103 104 105 106 107 108 109 110 111
BS15	122 123 124 125 126 127 149 150 151 152 153 154 176 194
BS16	34 35 36 37 38 40 96 105 111 112 113 114 115 116 202 224 228 229 231 232 235 236 237 238
BS17	4 5 6 7 63 135 136 141 142 143 144 145 146 147
BS18	43 46 47 48 49 50 51 52 54 55 79 80 81 85
BS19	26 27 28 29 45 141 186
BS20	20 22 26 28 47 48 49 50 54 55
BS21	9 58 59 60 61 66 67 71 72 106 107 108 109
BS22	161 162 163 165 210 215 216 217
BS23	9 58 59 60 61 66 67 71 72 106 107 108 109

Table S29. List of protein residues defining each binding site identified across the four independent 400 ns fdGaMD simulation replicates for system with PDB ID **5T4U**. In this system, the first residue of the simulated target (UniProtKB P55201) corresponds to M628 in the canonical sequence. Binding site corresponding to the crystallographic information available is marked with an asterisk (*).

BS	Protein Residues
BS1*	24 25 26 28 29 30 31 34 35 38 77 80 81 87
BS2	16 17 18 20 21 22 23 25 26 48 70 90 93 94 97 98 101
BS3	9 12 13 16 101 104 105 108

Table S30. List of protein residues defining each binding site identified across the four independent 400 ns fdGaMD simulation replicates for system with PDB ID **3E63**. In this system, the first residue of the simulated target (UniProtKB O60674) corresponds to D840 in the canonical sequence. Binding site corresponding to the crystallographic information available is marked with an asterisk (*).

BS	Protein Residues
BS1	106 107 209 210 211 212 213 216 252 253 254 255
BS2*	16 17 18 19 24 41 43 72 90 91 92 93 94 95 96 97 141 142 144 154 155
BS3	184 185 187 188 199 239 240 241 243 244 248 269 270 271 272
BS4	167 168 185 191 192 193 194 195 196 271 272 276
BS5	101 102 105 108 109 110 111 113 114 117 148 208
BS6	7 44 45 46 47 48 77 78 79 80 83 84 85 86 87
BS8	225 226 230 238 241 242 245 246 247 248
BS9	258 261 262 265 266 272 273 274 275 276 279 280 283 287
BS1	106 107 209 210 211 212 213 216 252 253 254 255

Table S31. List of protein residues defining each binding site identified across the four independent 400 ns fdGaMD simulation replicates for system with PDB ID **3NUN**. In this system, the first residue of the simulated target (UniProtKB O15530) corresponds to P71 in the canonical sequence. Binding site corresponding to the crystallographic information available marked with an asterisk (*).

**BS**	Protein Residues
BS1	3 4 41 43 56 57 58 60 61 64 65 75 87 89
BS2	1 2 64 67 68 69 70 71 72 73 74 75 89 154
BS3	68 69 70 71 121 276 277 278 279 280 281 282
BS4	94 97 100 101 104 106 107 108 111 115 143 147 285 286 287 288
BS5	43 44 45 48 49 54 57 58 61 78 80 81 82 83 84 85 87
BS6	98 99 101 102 105 106 107 108 112 204 205 206 207 208 236
BS7	59 132 134 158 159 160 161 162 163 164 165 166 167 168 169 170 171 172 173 174 183 187 188 189 190 191
BS8	110 111 113 114 271 273 274 275 276 280 283 284 285 286 287
BS9*	16 18 19 24 26 28 37 38 39 41 56 60 64 73 75 89 90 91 92 93 94 95 99 142 152 153 154 155
BS10	34 36 37 38 39 74 76 89 90 91 92 144 145 146
BS11	245 248 253 254 255 260 265
BS12	123 124 127 128 132 134 160 161 162 163 168 189 191 192 194 195 256 257 258 2559 263
BS13	203 207 209 210 211 212 213 223 228 229 230 231 232 233
BS14	114 146 147 148 281 282 283 284 285 288
BS15	23 24 41 43 47 48 53 56 57 135 155 156 174 175
BS16	181 182 184 185 186 188 189 190 225 249 250 251 252 253
BS17	55 56 58 59 62 134 155 156 157 158 163 164 165 166 170 171 172 183 187
BS18	59 62 63 66 67 68 125 126 129 131 158 159 160 161 162
BS19	16 18 28 37 38 39 91 92 93 94 95 96

Root-Mean Square Deviation (for the selected trajectories)

This section presents the RMSD values computed over the final 100 ps of simulation of the validation systems studied.

Table S32. Root-Mean Square Deviation (RMSD) analysis for the final selected ligand trajectory of the validation systems in sets II and III, identified by their corresponding PDB IDs. Mean and standard deviation values depicted were obtained from the last 100 ps (from 400 ns fdGaMD) of the individual ligand trajectory, employing the corresponding X-ray structure as reference for the calculations. RMSD analysis performed for the original conventional approach at the last 100 ps of 400 ns fdMD (†) for validation set-II, including the RMSD results obtained for system 4FU8 at the last 100 ps prior unbinding (at 375 ns, conventional fdMD; ‡), have also been included. RMSD analysis of the last 100 ps of the equilibrium (native) conformation of system 5YE8 has been also considered for the analysis (§). RMSD values for the last 100 ps of simulation for the reactive trajectories of system 4CR5 identified at the 12 (400ns fdGaMD) replicates experience for system 4CR5 with native (*), flipped (**), and backwards (⁂) conformations have also been included in the analysis.

	PDB ID	Mean RMSD (Å)	Std. Dev. RMSD (Å)
SET II	4FUD [52]	1.0290<br>3.4062†	0.3024<br>0.0547†
	4FU8 [52]	1.4164<br>41.6424†<br>12.9275‡	0.2581<br>2.03572†<br>0.0922‡
	4FUD [52]	0.8286<br>4.1140†	0.2928<br>0.1555†
SET III	1I06 [54]	2.7657	0.0991
	5YE8 [55]	2.2281<br>1.6832§	0.1567<br>0.2245§
	1W7H [58]	1.1858	0.4369
	4CR5 [59]	5.0460<br>1. 2456*<br>2.2703**<br>4.6814⁂	0.4894<br>0.3485*<br>0.0752**<br>0.0998⁂
	5T4U [60]	1.3290	0.3452
	3E63 [61]	0.7995	0.1634
	3NUN [62]	0.4891	0.1877

Additional results from the trajectory analysis

Supplementary figures included in this section support and complement the findings reported in the main manuscript.

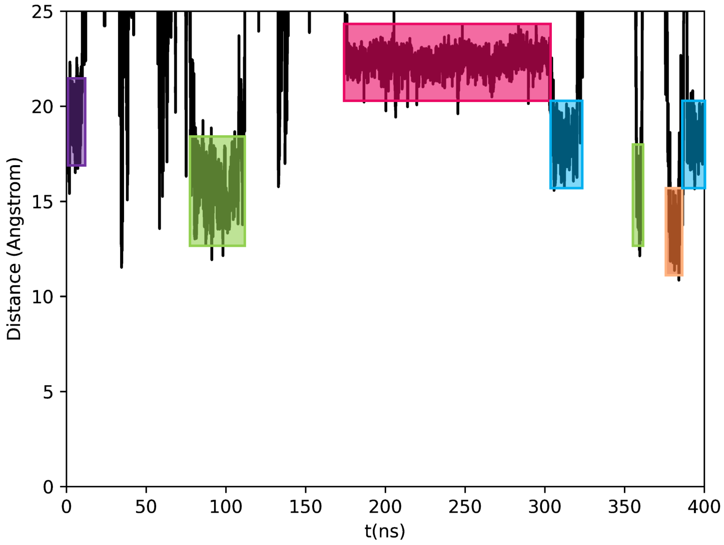

Figure S2. Representation of the time evolution of the center of masses distance between the crystallographic and the simulated ligand, along a representative trajectory extracted from a 400 ns of GaMD simulation replicate for the urokinase system 1F5K (set-II). Colored boxes highlight alternative binding sites explored during the simulation, distinct from the orthosteric site. Within this representation, distances exceeding 25 Å indicate the fragment is in bulk solvent.

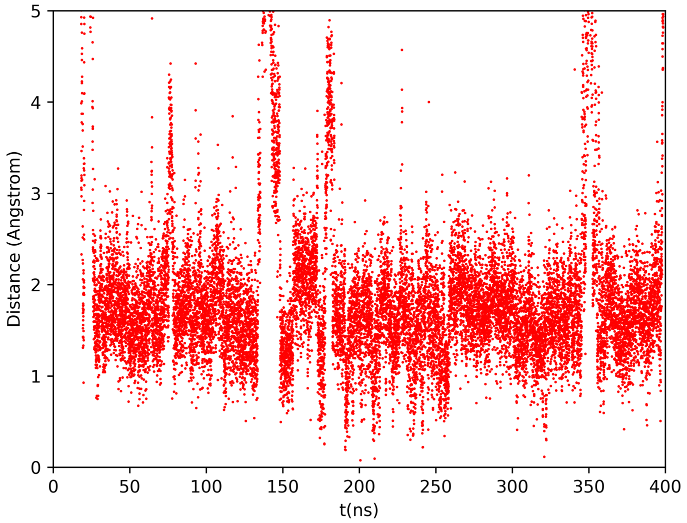

Figure S3. Scatter representation of the time evolution of the distance between the center of masses of the simulated and reference crystallographic fragment position, computed for a discarded trajectory from the 400 ns of conventional fdMD simulations performed for the urokinase system 4FU8 (set-II), interacting at the experimental binding site of the protein.

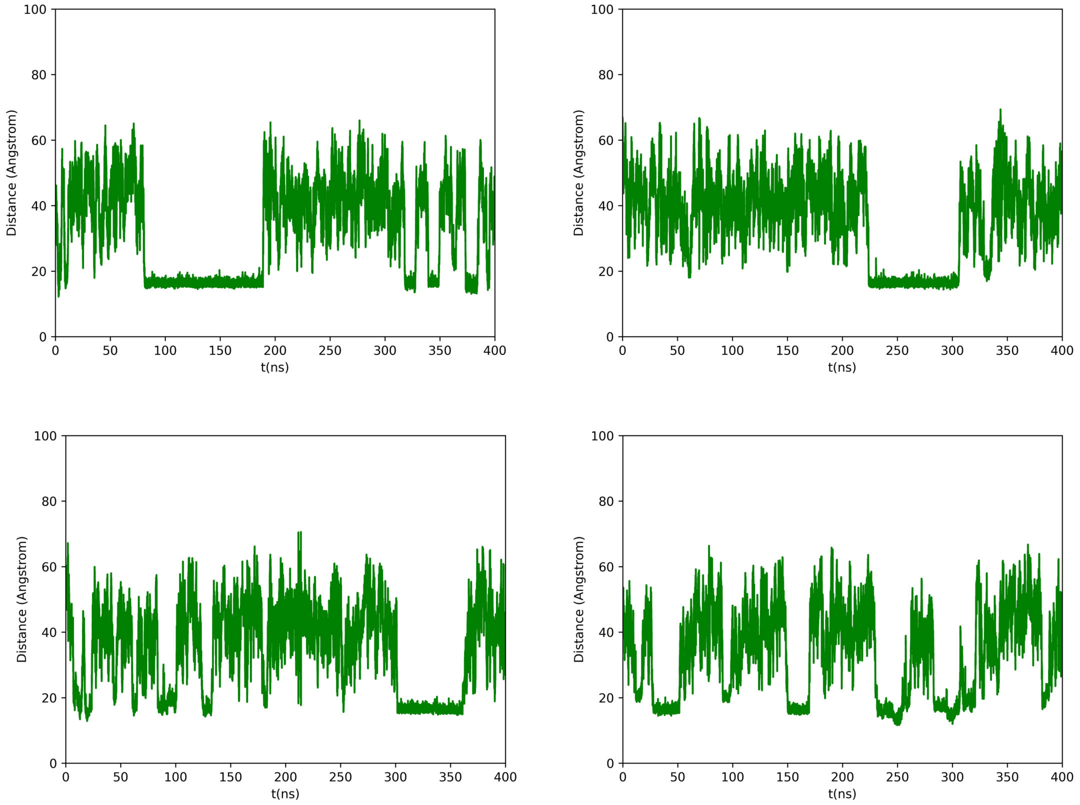

Figure S4. Representation of the time evolution of the distance between the center of masses of the simulated ligand and the target protein, computed for different "non-reactive" individual ligand trajectories of system with PDB ID 1I06 (set-III), which exhibited recurrent association and dissociation events during the simulations. Distances around 20 Å show ligand-receptor interactions, while higher distances correspond to the fragment in the bulk.

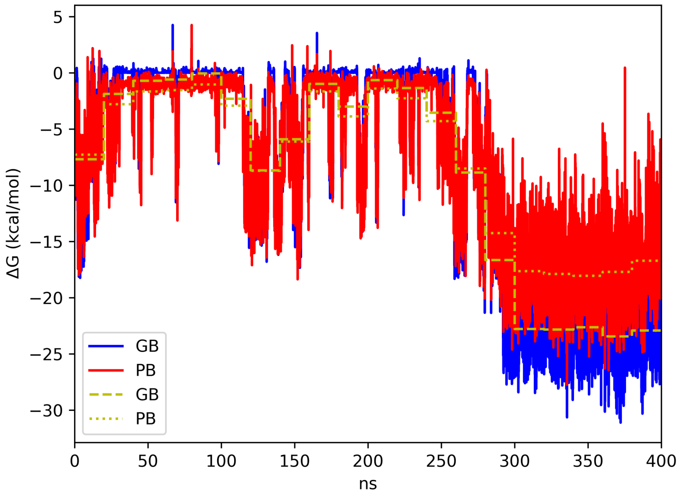

Figure S5. Free binding energy profile of the "non-reactive" trajectory corresponding to the experimental binding site (pre-pocket pose) identified at one of the 4 fdGaMD replicates performed for system 4CR5. Generalized-Born MMGBSA (blue) and Poisson-Boltzmann MMPBSA (red) energy estimations over time, and averaged values at every 20 ns represented as yellow dashed lines. Good, converged results identified for the last 110 ns of interaction (RT).

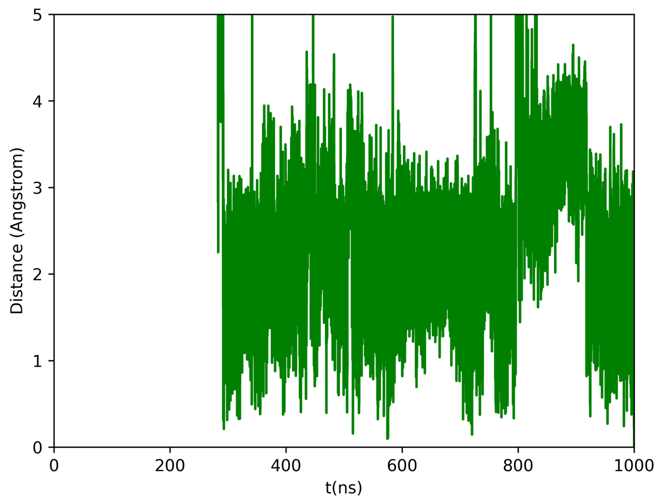

Figure S6. Time evolution of the distance between the ligand central atom and the reference position from the last snapshot of the "reactive" **extended 1 μs** fdGaMD simulation replicates of system **4CR5**. Except for a brief recovered fluctuation, the fragment conformation was maintained in the same pre-pocket flipped binding mode, as previously identified at the 400 ns analysis.

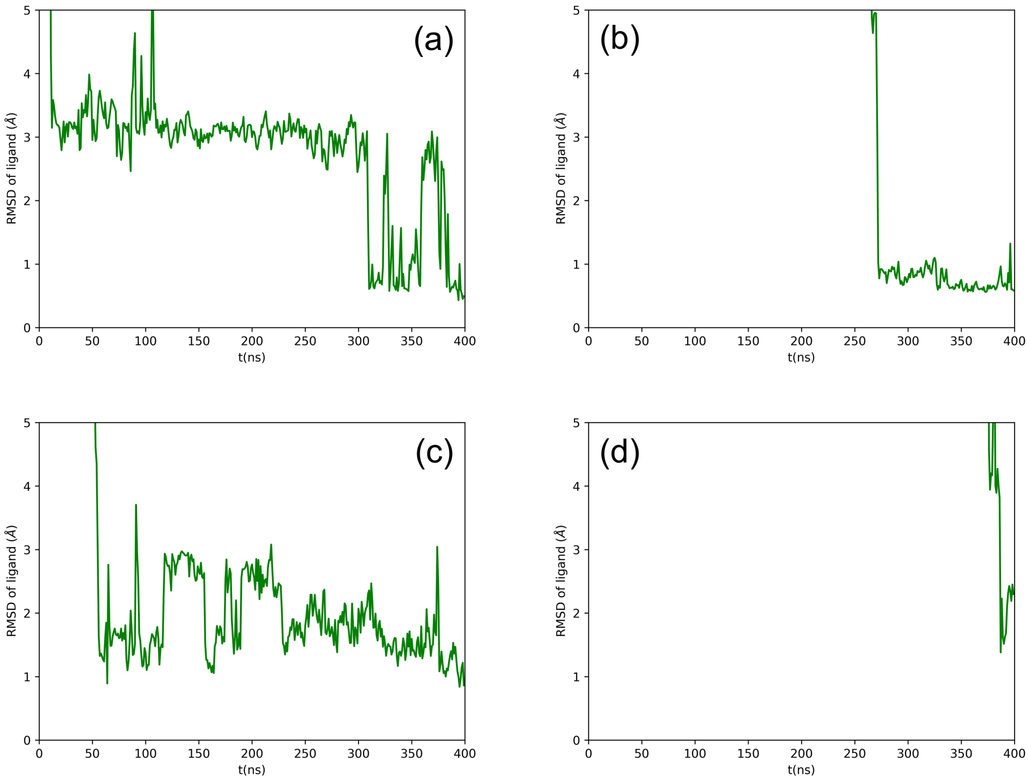

Figure S7. Representation of the ligand RMSD evolution for the four "reactive" trajectories of the experimental binding site from the **12** independent **400 ns** fdGaMD simulation replicates from system **4CR5**, corresponding to the native (a), backwards (b) and flipped (c & d) binding mode conformations of the ligand. Hydrogen atoms have been omitted for RMSD calculations. All conformations exhibited low RMSD values (~1–3 Å), consistent with the small ligand size and experimental binding site proximity. Fluctuations observed, particularly for the native conformation (a), highlight the existence of pre-binding states participating in ligand accommodation to stablish optimal binding interactions. The last frame of the simulation has been employed as reference for the RMSD calculations.

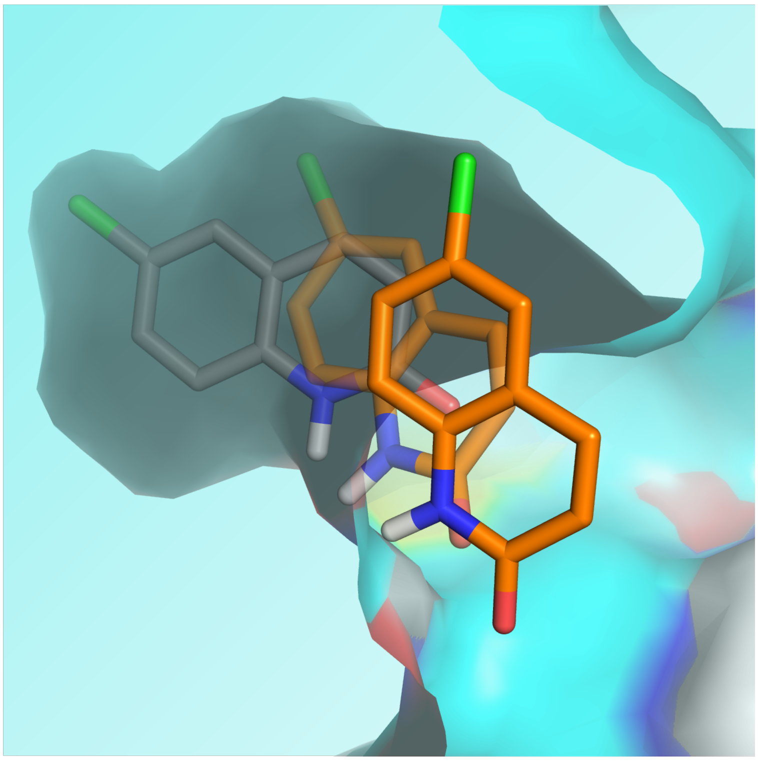

Figure S8. Representation of 2 "pre-pocket" binding mode states identified at the experimental site for system with PDB ID **4CR5** for the **48** independent replicates of **100 ns** (depicted in orange) fdGaMD simulation length, superposed to its crystallographic X-ray structure (depicted in grey), with the simulated protein surface shown in cyan. Non-polar hydrogen atoms are omitted for clarity. As observed, native interactions are not reproduced in less simulation time replicates.

Scripts

Within this section find the computational details regarding principal axis orientation.

```
cpptraj > cpptraj_system_principal.out << EOF
   parm system.top
   trajin system.pdb
   principal dorotation out rotation.out
   trajout system_principal.pdb
   run
   EOF
```

Supporting Figure S9. Example of the principal axis orientation script used along the fdGaMD preparation protocol. Based on the calculation of the principal moments of inertia through the diagonalization of the inertial tensor matrix of the system, the approach is aimed at applying rotation and coordinates alignment. The alignment is done along the longest axis (higher associated eigenvalue), that we finally set along the Z-axis direction. To align the ligand along its principal axis for the preparation of the ligand solvation boxes to efficiently solvate the protein, also in its principal axis orientation, we used the principal command in CppTraj module from AMBER22.[73]
